# Combined glycoprotein IIb/IIIa inhibitor therapy with ticagrelor for patients with acute coronary syndrome

**DOI:** 10.1371/journal.pone.0246166

**Published:** 2021-02-02

**Authors:** Zhi-Jiang Xie, Shuan-Li Xin, Chao Chang, Hai-Jing Zhou, Xiu-Feng Zhao, Feng-Hui Jiao, Chuan Chen, Tao Li

**Affiliations:** 1 Department of Cardiology, Handan First Hospital, Handan, China; 2 Department of Gynecology, Handan Central Hospital, Handan, China; Medizinische Universitat Graz, AUSTRIA

## Abstract

This study was to compare the efficacy and safety of combined glycoprotein IIb/IIIa inhibitor (GPI) and ticagrelor versus ticagrelor in patients with acute coronary syndrome (ACS). An observational study was conducted using the Improving Care for Cardiovascular Disease in China-ACS project. Totally, 13,264 patients with ACS and received combination therapy or ticagrelor therapy were analyzed. The primary outcome was the composite of major cardiovascular events (MACE: all-cause mortality, myocardial infarction [MI], stent thrombosis, cardiogenic shock, and ischemic stroke), and secondary outcomes included all-cause mortality, MI, stent thrombosis, cardiogenic shock, and ischemic stroke. The multivariable adjusted analysis indicated that combination therapy was associated with an increased risk of major cardiovascular events (MACE) (*P* = 0.001), any bleeding (*P*<0.001), and major bleeding (*P* = 0.005). Moreover, the multivariable adjusted for propensity score-matched (PSM) analysis suggested that combination therapy produced additional risk of MACE (*P* = 0.014), any bleeding (*P*<0.001), and major bleeding (*P* = 0.005). Moreover, PSM analysis suggested that combination therapy was associated with greater risk of stent thrombosis (*P* = 0.012) and intracranial bleeding (*P* = 0.020). Combined GPI and ticagrelor therapies did not have any beneficial effects on MACE, stent thrombosis, intracranial bleeding, any bleeding, or major bleeding.

## Introduction

Platelet aggregation is an independent predictor of adverse cardiac events in patients after percutaneous coronary intervention (PCI) [1]. Ticagrelor is a potent P2Y_12_ adenosine diphosphate receptor antagonist and produces a faster onset and consistent and reversible antiplatelet effect with fewer adverse events than existing P2Y_12_ receptor antagonists [2]. The PLATO trial found that patients with acute coronary syndrome (ACS) who received ticagrelor had significantly reduced risk of composite cardiovascular outcome compared to patients who received clopidogrel without an increase in overall major bleeding events [3]. However, a prior pharmacodynamic study indicated that the rate of high on-treatment platelet reactivity occurs at 2 hours in unstable angina patients receiving ticagrelor, which suggests that ticagrelor does not achieve optimal platelet inhibition during PCI [4]. Therefore, glycoprotein IIb/IIIa inhibitors (GPIs) should be used for patients undergoing PCI to achieve optimal platelet inhibition [5].

Previous studies have shown that intravenous GPIs produce a clinical benefit in patients with ACS [6–8]. Although concomitant GPIs and P2Y_12_ inhibitors can improve ischemic outcomes, whereas the risk of bleeding is significantly increased [9–11]. Currently, the American College of Cardiology/American Heart Association guidelines has provided the class I recommendation for patients with ACS to not receive P2Y_12_ inhibitors and the class IIa recommendation for patients at high risk to receive clopidogrel pretreatment [12]. However, whether concomitant GPI and ticagrelor use provides superior clinical benefit with respect to subsequent adverse events over ticagrelor remains controversial. We therefore compared the efficacy and safety of GPI plus ticagrelor versus ticagrelor in patients with ACS in the Improving Care for Cardiovascular Disease in China-ACS (CCC-ACS) project.

## Methods

### Patient population

**Fig 1:**
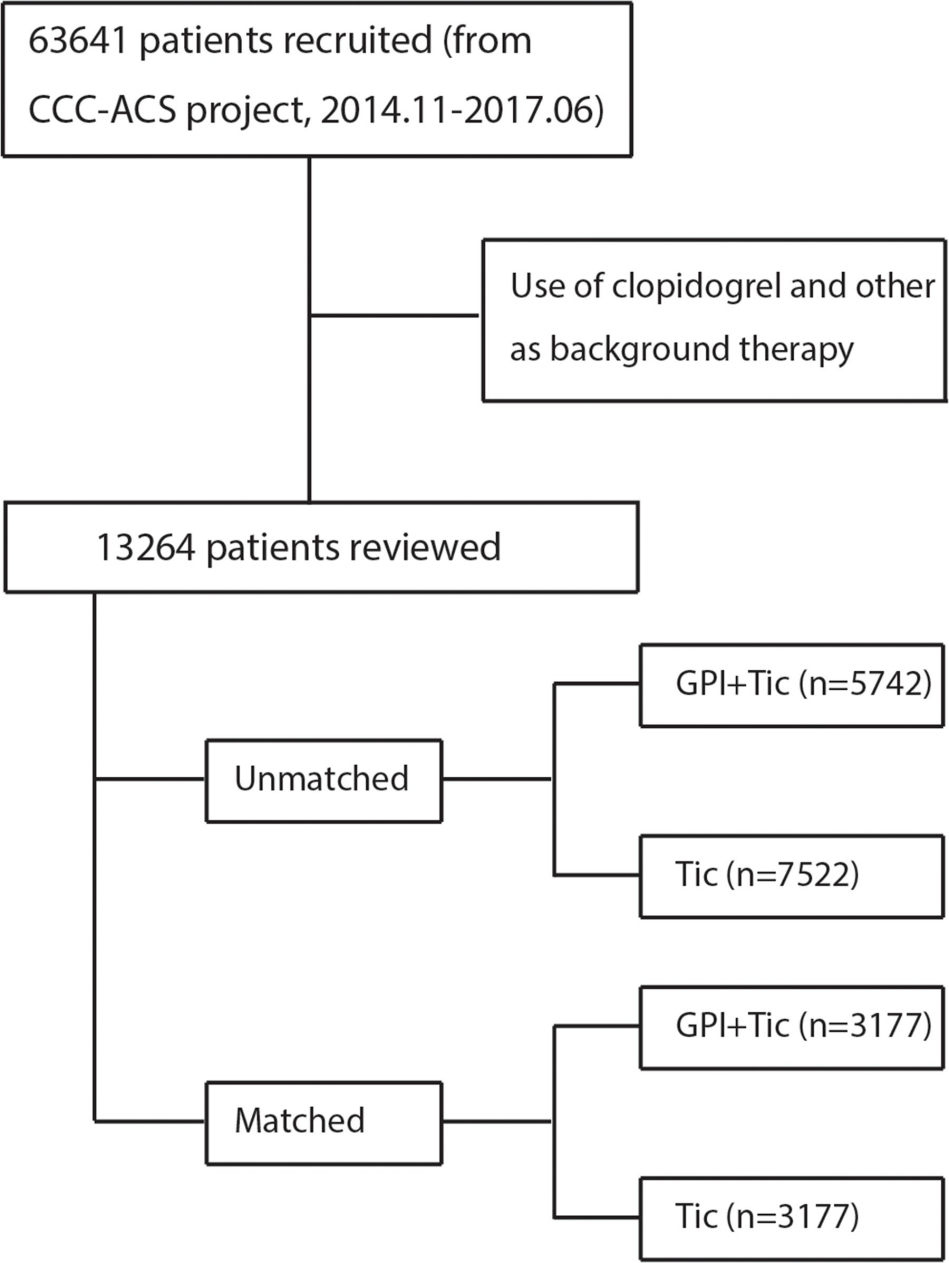
The study population.

This study used data from the CCC-ACS project, which is a nationwide registry and quality improvement study with an ongoing database that focuses on quality of care for ACS. The details of the CCC-ACS project have been previously described [13, 14]. Patients’ medical records were collected by trained data abstractors at the participating hospitals through a standard web-based data collection platform (Oracle Clinical Remote Data Capture, Oracle). The CCC-ACS project enrolled 63,641 patients with ACS at 150 Chinese hospitals between November 1, 2014, and June 30, 2017. The data cleaning was conducted systematically, and the query data for invalid and illogical values through searching for outliers in continuous data distributions. All patients’ records were analyzed in a fully anonymized and de-identified manner, and the patients’ personal information were not accessed by researcher. Institutional review board approval was granted for this research by the ethics committee of Beijing Anzhen Hospital, Capital Medical University, and informed consent was not required. For the purpose of this study, patients who received clopidogrel were excluded; 13,264 patients who received ticagrelor within 24 hours of first medical contact did not switch drugs were included. Of the 13,264 enrolled patients, 5,742 received GPI and the remaining 7,522 did not (Fig 1).

### Definitions and outcomes

The primary outcome of this study was the composite of major cardiovascular events (MACE: all-cause mortality, myocardial infarction [MI], stent thrombosis, cardiogenic shock, and ischemic stroke), with the secondary outcomes including all-cause mortality, cardiac death, MI, stent thrombosis, cardiogenic shock, and ischemic stroke. Safety outcomes included any bleeding (intracranial bleeding, gastrointestinal bleeding, bleeding requiring surgical intervention, transfusion with overt bleeding, and CABG bleeding) [15], and major bleeding (intracranial bleeding, retroperitoneal hemorrhage, >4 g/dL decrease in hemoglobin, bleeding requiring surgical intervention, and transfusion with overt bleeding). All-cause mortality was defined as cardiac death and non-cardiac death with definitive cause. MI was defined as elevated cardiac enzymes with ischemic symptoms or electrocardiography findings. Stent thrombosis was defined as a thrombus formed in the stent implanted into the coronary artery. Cardiogenic shock was defined as severely impaired cardiac function, causing a marked reduction in cardiac output and severe acute peripheral circulatory failure. Stroke was divided into ischemic stroke and intracranial bleeding, which was defined as a neurological deficit caused by an acute focal injury of the central nervous system of vascular cause. The definition of bleeding events have already described in a previous study [16].

### Statistical analysis

The characteristics of the enrolled patients and outcomes were analyzed comparing GPI plus ticagrelor to ticagrelor. Continuous data are presented as mean and standard deviation and compared using the *t* test or Wilcoxon rank-sum test. Categorical data are shown as the number of events and percentages and compared using the chi-square test. Odds ratios (ORs) with corresponding 95% confidence intervals (CIs) were calculated using univariate and multiple logistic regression models. Candidate adjusted variables appearing to be related by the univariate analysis with p<0.10 were entered in the step-wise method for select predictors of primary outcomes and safety outcomes. Moreover, the propensity score-matched (PSM) method was used to adjust for any imbalance in demographic and clinical characteristics to avoid undue influences of confounding factors. The propensity score provides the probability of each patient being treated with GPI plus ticagrelor or ticagrelor based on the predicted probabilities from a multiple logistic regression analysis. The matched data set that was obtained employed the variable ratio and parallel and pair-wise nearest neighbor matching methods to avoid significant data loss from the total cohort. A standardized mean difference of <10% for matching variables was considered an appropriate balance between the groups. Categorical data from PSM analysis were compared using the McNemar’s or Bowker’s test, and the paired t test was employed for continuous data. The risks of investigated outcomes were evaluated using a logistic regression model to calculate the effect estimate and presented as ORs with corresponding 95% CIs. Furthermore, stratified analyses were conducted for MACE, any bleeding, and major bleeding based on unmatched data and data matched according to age, sex, renal insufficiency, diabetes, weight, and disease status. The reported p values are 2-sided, and *P*<0.05 was regarded as statistically significant. Statistical analyses were performed using SPSS 23.0 (IBM) and STATA 12.0 (StataCorp).

## Results

### Baseline characteristics

The characteristics of the unmatched patients in the GPI plus ticagrelor group (n = 5,742) and ticagrelor group (n = 7,522) are shown in Table 1. There were significant differences between combined therapy and ticagrelor therapy for sex, age, smoking, previous MI, previous PCI, heart failure history, hypertension, peripheral vascular disease (PVD) history, chronic obstructive pulmonary disease, renal failure history, type of ACS, SBP, Killip class, cardiogenic shock, heart failure, sudden cardiac arrest, PCI, culprit vessel, stent implantation, statins, β-blocker, ACEI, ARB, aldosterone receptor antagonist, warfarin, anticoagulant drug use, hemoglobin, serum creatinine, FBG, INR, and TC. Moreover, there were no significant differences in previous CABG, AF history, DM, dyslipidemia, stroke history, heart valve surgery history, heart rate, DBP, aspirin use, TG, HDL, and LDL between the 2 groups. Three thousand one hundred and seventy-seven patients in the GPI plus ticagrelor group and 3,177 patients in the ticagrelor group were matched using a variable 1:1 matching after PSM analysis of the whole cohort (Table 1). Mostly, the patient characteristics between the two groups were balanced, but differences in patients’ mean age, type of ACS, and admission due to sudden cardiac arrest were statistically significant.

**Table 1:** Baseline characteristics of included patients.

Variable	Unmatched			Propensity Score-Matched		
	Ticagrelor (n = 7522)	Ticagrelor+GPI (n = 5742)	P value	Ticagrelor (n = 3177)	Ticagrelor+GPI (n = 3177)	P value
Sex (Female)	1740 (23.1)	1030 (17.9)	< 0.001	621 (19.5)	652 (20.5)	0.331
Age (years)	62.04±12.44	59.54±11.83	< 0.001	60.26±11.97	61.2±11.06	0.001
Smoking	3519 (46.8)	2994 (52.1)	<0.001	1618 (50.9)	1600 (50.4)	0.652
Previous MI	462 (6.1)	286 (5.0)	0.004	165 (5.2)	162 (5.1)	0.865
Previous PCI	505 (6.7)	331 (5.8)	0.026	190 (6.0)	193 (6.1)	0.874
Previous CABG	24 (0.3)	14 (0.2)	0.422	7 (0.2)	6 (0.2)	0.681
AF history	121 (1.6)	80 (1.4)	0.314	48 (1.5)	49 (1.5)	0.919
HF history	74 (1)	27 (0.5)	0.001	17 (0.5)	17 (0.5)	1.000
Hypertension	3849 (51.2)	2762 (48.1)	<0.001	1604 (50.5)	1648 (51.9)	0.269
DM	1601 (21.3)	1182 (20.6)	0.327	680 (21.4)	662 (20.8)	0.580
Dyslipidemia	452 (6.0)	375 (6.5)	0.218	208 (6.5)	180 (5.7)	0.142
Stroke history	609 (8.1)	457 (8.6)	0.773	262 (8.2)	245 (7.7)	0.431
PVD history	57 (0.8)	25 (0.4)	0.019	20 (0.6)	15 (0.5)	0.397
COPD	82 (1.1)	40 (0.7)	0.019	20 (0.6)	27 (0.8)	0.305
Renal failure history	88 (1.2)	33 (0.6)	<0.001	16 (0.5)	14 (0.4)	0.714
Heart valve surgery history	6 (0.1)	2 (0.03)	0.492	1 (0.0)	0 (0.0)	0.239
Type of ACS						
STEMI	5394 (71.7)	4921 (85.7)	<0.001	2466 (77.6)	2718 (85.6)	<0.001
NSTEMI	1553 (20.6)	652 (11.4)		528 (16.6)	410 (12.9)	
UAP	481 (6.4)	521 (2.1)		183 (5.8)	49 (1.5)	
Heart rate	78.24±16.31	77.76±16.23	0.094	78.03±15.58	77.70±15.56	0.390
SBP (mmHg)	129.45±23.97	127.82±24.32	<0.001	129.11±23.70	129.95±23.93	0.163
DBP (mmHg)	78.24±16.31	78.42±15.31	0.284	78.75±14.72	78.86±14.94	0.761
Killip class						
I	5131 (71.7)	4159 (75.65)	<0.001	2405 (75.7)	2420 (76.2)	0.427
II	1476 (20.6)	940 (17.10)		605 (19.0)	589 (18.5)	
III	270 (3.8)	140 (2.55)		84 (2.6)	70 (2.2)	
IV	279 (3.9)	259 (4.71)		83 (2.6)	98 (3.1)	
Cardiogenic shock	259 (3.4)	285 (5.0%)	<0.001	108 (3.4)	85 (2.7)	0.093
Heart failure	584 (7.8)	413 (7.2%)	<0.001	192 (6.0)	183 (5.8)	0.683
Sudden cardiac arrest	159 (2.1)	177 (3.1%)	<0.001	61 (1.9)	33 (1.0)	0.004
PCI	5853 (77.8%)	5349 (93.2%)	<0.001	3106 (97.8)	3116 (98.1)	0.379
Culprit vessel						
LM	71 (1.2)	56 (1.0)	<0.001	31 (1.0)	26 (0.8)	0.196
LAD	2609 (43.6)	2487 (46.2)		1444 (45.5)	1489 (46.9)	
LCX	888 (14.8)	675 (12.5)		479 (15.1)	413 (13.0)	
RCA	2236 (37.3)	2034 (37.8)		1140 (35.9)	1153 (36.3)	
SVG	13 (0.2)	11 (0.2)		9 (0.3)	8 (0.3)	
Other	170 (2.8)	118 (2.2)		74 (2.3)	88 (2.8)	
Stent implantation	5358 (60.1%)	3550 (61.8%)	<0.001	2699 (85.0)	2683 (84.5)	0.577
Aspirin	7367 (97.9)	5641 (98.2)	0.373	3125 (98.4)	3120 (98.2)	0.689
Statins	7151 (95.1)	5560 (96.8)	<0.001	3093 (97.4)	3079 (96.9)	0.292
β-blocker	4257 (56.6)	3417 (59.5)	<0.001	1779 (56.0)	1807 (56.9)	0.479
ACEI	822 (10.9)	510 (8.9)	<0.001	307 (9.7)	329 (10.4)	0.358
ARB	2932 (39)	2130 (37.1)	<0.001	1260 (39.7)	1234 (38.8)	0.511
Adosterone receptor antagonist	1366 (18.2)	968 (16.9)	<0.001	506 (15.9)	512 (16.1)	0.837
Warfarin	53 (0.7)	12 (0.2)	<0.001	8 (0.3)	8 (0.3)	1.000
Anticoagulant therapy	5773 (76.7)	4923 (85.7)	<0.001	2638 (83.0)	2623 (82.6)	0.618
Hemoglobin (g/L)	137.64±20.44	140.99±19.97	<0.001	140.45±19.44	139.69±18.59	0.110
Serum creatinine (umol/L)	83.75±55.65	81.20±41.95	0.004	80.45±49.21	80.17±43.03	0.808
FBG (mmol/L)	7.15±3.22	7.083±3.07	0.021	7.15±3.17	7.02±2.96	0.081
INR	1.12±0.84	1.07±0.53	0.001	1.09±0.58	1.08±0.49	0.569
TC (mmol/L)	4.52±1.27	4.60±1.22	<0.001	4.59±1.21	4.57±1.19	0.930
TG (mmol/L)	1.78±1.32	1.80±1.45	0.304	1.78±1.29	1.79±1.43	0.727
HDL (mmol/L)	1.11±0.45	1.11±0.41	0.824	1.11±0.45	1.11±0.41	0.349
LDL (mmol/L)	2.80±1.01	2.87±1.00	0.065	2.86±1.00	2.85±0.99	0.694

*MI: myocardial infarction; PCI: percutaneous coronary intervention; CABG: coronary artery bypass grafting; AF: atrial fibrillation; HF: heart failure; DM: diabetes mellitus; PVD: peripheral vascular disease; COPD: chronic obstructive pulmonary disease; SBP: systolic blood pressure; DBP: diastolic blood pressure

### Univariable analysis

The in-hospital outcomes within 15 days of hospitalization between the GPI plus ticagrelor group and the ticagrelor group from unmatched and matched cohorts are presented in Table 2. In the unmatched cohort, we noted that patients who received combination therapy had a greater risk of MACE (*P* = 0.012), stent thrombosis (*P*<0.001), cardiogenic shock (*P*<0.001), any bleeding (*P*<0.001), and major bleeding (*P* = 0.003); however, it was associated with a lower risk of all-cause mortality (*P* = 0.019) and ischemic stroke (*P* = 0.045). Moreover, there were no significant differences between groups for the risk of cardiac death (*P* = 0.091), MI (*P* = 0.149), intracranial bleeding (*P* = 0.100), gastrointestinal bleeding (*P* = 0.058), retroperitoneal hemorrhage (*P* = 0.473), >4 g/dL decrease in hemoglobin (*P* = 0.095), bleeding requiring surgical intervention (*P* = 0.502), transfusion with overt bleeding (*P* = 0.458), or CABG bleeding (*P* = 0.067). Similarly, after PSM analysis, we noted that patients receiving combination therapy had a greater risk of stent thrombosis (OR: 4.347; 95% CI: 1.238–15.269; *P* = 0.012), any bleeding (OR: 1.607; 95% CI: 1.240–2.082; *P*<0.001), major bleeding (OR: 1.540; 95% CI: 1.122–2.114; *P* = 0.008), and intracranial bleeding (OR: 2.317; 95% CI: 1.176–4.566; *P* = 0.020), but no significant difference in the risk of MACE (*P* = 0.126), all-cause mortality (*P* = 0.150), cardiac death (*P* = 0.081), MI (*P* = 0.637), cardiogenic shock (*P* = 0.120), ischemic stroke (*P* = 0.404), gastrointestinal bleeding (*P* = 0.058), retroperitoneal hemorrhage (*P* = 0.985), >4 g/dL decrease in hemoglobin (*P* = 0.453), bleeding requiring surgical intervention (*P* = 0.466), transfusion with overt bleeding (*P* = 0.589), or CABG bleeding (*P* = 0.571).

**Table 2:** In-hospital outcomes within 15 days after hospitalization.

Outcomes	Unmatched				PSM cohort			
	Ticagrelor (n = 7522)	Ticagrelor+GPI (n = 5742)	OR and 95%CI	P value	Ticagrelor (n = 3177)	Ticagrelor+GPI (n = 3177)	OR and 95%CI	P value
MACE	342	316	1.223 (1.045–1.430)	0.012	96	118	1.238 (0.941–1.628)	0.126
All-cause mortality	160	90	0.733 (0.565–0.951)	0.019	35	24	0.683 (0.406–1.151)	0.150
Cardiac death	154	87	0.847 (0.233–3.085)	0.091	34	21	0.615 (0.356–1.062)	0.081
Myocardial infarction	26	12	0.604 (0.304–1.198)	0.149	10	8	0.799 (0.315–2.028)	0.637
Stent thrombosis	6	26	5.698 (2.344–13.582)	<0.001	3	13	4.347 (1.238–15.269)	0.012
Cardiogenic shock	227	244	1.426 (1.186–1.714)	<0.001	67	86	1.375 (0.935–1.784)	0.120
Ischemic stroke	20	6	0.392 (0.157–0.978)	0.045	4	2	0.500 (0.091–2.730)	0.404
All bleeding	310	331	1.423 (1.214–1.668)	<0.001	97	153	1.607 (1.240–2.082)	<0.001
Major bleeding	234	234	1.323 (1.100–1.591)	0.003	65	99	1.540 (1.122–2.114)	0.008
Intracranial bleeding	40	45	1.470 (0.960–2.250)	0.100	12	28	2.317 (1.176–4.566)	0.020
Gastrointestinal bleeding	81	83	1.347 (0.990–1.833)	0.058	28	44	1.579 (0.981–2.543)	0.058
Retroperitoneal hemorrhage	3	1	0.437 (0.045–4.198)	0.473	0	1	NA	0.985
The decrease in hemoglobin > 4g/dL	163	150	1.211 (0.968–1.516)	0.095	44	57	1.164 (0.782–1.732)	0.453
Bleeding requiring surgical intervention	32	29	1.188 (0.719–1.966)	0.502	10	7	0.699 (0.266–1.840)	0.466
Transfusion with overt bleeding	41	26	0.830 (0.507–1.358)	0.458	14	17	1.215 (0.598–2.470)	0.589
CABG bleeding	9	1	0.145 (0.018–1.148)	0.067	2	1	0.500 (0.045–5.515)	0.571

### Multivariable analysis

The results of multivariable analysis adjusted for the risk of MACE, any bleeding, and major bleeding in unmatched and matched cohorts are presented in S1 File. The results from the unmatched cohort indicated that patients in the GPI plus ticagrelor group had an increased risk of MACE (OR: 1.669; 95% CI: 1.249–2.231; *P* = 0.001), any bleeding (OR: 1.605; 95% CI: 1.259–2.046; *P*<0.001), and major bleeding (OR: 1.518; 95% CI: 1.137–2.027; *P* = 0.005). Similarly, the multivariate-adjusted PSM analysis found a greater risk in the GPI plus ticagrelor group of MACE (OR: 1.505; 95% CI: 1.086–2.084; *P* = 0.014), any bleeding (OR: 1.631; 95% CI: 1.244–2.139; *P*<0.001), and major bleeding (OR: 1.616; 95% CI: 1.158–2.257; *P* = 0.005). Moreover, the multivariable analysis from the unmatched cohort found patients in the GPI plus ticagrelor group were not associated with the risk of all-cause mortality (*P* = 0.725), cardiac death (*P* = 0.865), MI (*P* = 0.285), and ischemic stroke (*P* = 0.208), while it was associated with an increased risk of stent thrombosis (*P* = 0.003), and cardiogenic shock (*P* = 0.003). Finally, the multivariate-adjusted PSM analysis suggested patients in the GPI plus ticagrelor group was associated with an increased risk of stent thrombosis (*P* = 0.010), and cardiogenic shock (*P* = 0.011), whereas no significant differences between groups for the risk of all-cause mortality (*P* = 0.348), cardiac death (*P* = 0.255), MI (*P* = 0.696), and ischemic stroke (*P* = 0.348).

### Stratified analyses

Analyses for MACE, any bleeding, and major bleeding stratified based on age, sex, renal insufficiency, diabetes, weight, and disease status in unmatched and matched cohorts were conducted and listed in Table 3. In the unmatched cohort, we noted that the risk of MACE was significantly increased after combination therapy when patients were less than 75.0 years of age, were male, had renal insufficiency, were not diabetic, weighed greater than >60.0 kg, and had STEMI. Although the risk for any bleeding in patients receiving combination therapy was significantly increased in mostly subsets, we noted no significant differences between treatment groups when patients had renal insufficiency or diabetes. There were no significant differences between the groups for the risk of major bleeding when the patients were over 75.0 years of age, irrespective of renal insufficiency, diabetes, weight, or NSTEMI/UAP. Moreover, although the overall analysis indicated that combination therapy was associated with an increased risk of MACE, this significant difference was not observed in all subsets. Patients receiving combination therapy did not have any detrimental impact on any bleeding when patients had diabetes or weighed <60.0 kg. Finally, the risk of major bleeding was significantly increased when patients were under 75.0 years of age, had renal insufficiency, were not diabetic, weighed >60.0 kg, or had STEMI.

**Table 3:** Stratified analyses.

Outcomes	Variable	Group	Unmatched						Matched					
			Ticagrelor (n = 7522)		Ticagrelor+GPI (n = 5742)		OR and 95%CI	P value	Ticagrelor (n = 3177)		Ticagrelor+GPI (n = 3177)		OR and 95%CI	P value
MACE	Age (years)	≥75	127	1150	76	592	1.211 (0.643–2.280)	0.554	24	404	28	409	1.164 (0.662–2.044)	0.598
		< 75	215	5987	239	4805	1.706 (1.220–2.386)	0.002	72	2733	90	2768	1.261 (0.921–1.727)	0.149
	Sex	Male	232	5550	223	4489	1.760 (1.243–2.491)	0.001	67	2489	83	2442	1.263 (0.911–1.751)	0.162
		Female	110	1630	93	937	1.504 (0.838–2.701)	0.172	29	592	35	617	1.158 (0.699–1.919)	0.569
	Renal insufficiency	Yes	135	941	116	653	2.536 (1.455–4.422)	0.001	37	303	53	346	1.254 (0.802–1.962)	0.321
		No	125	3811	131	3343	1.352 (0.873–2.094)	0.177	41	1861	47	1961	1.088 (0.712–1.662)	0.697
	Diabetes	Yes	89	1512	67	1115	1.586 (0.766–3.283)	0.241	27	653	26	636	0.989 (0.517–1.713)	0.968
		No	253	5668	249	4311	1.753 (1.259–2.440)	0.001	69	2428	92	2423	1.336 (0.973–1.835)	0.073
	Weight (kg)	≥ 60	145	3653	135	2926	1.655 (1.078–2.541)	0.021	30	925	34	981	1.069 (0.649–1.760)	0.974
		< 60	81	1245	70	730	1.701 (0.838–3.453)	0.141	15	314	22	273	1.687 (0.858–3.317)	0.130
	Disease status	NSTEMI/UAP	72	1962	29	744	1.811 (0.812–4.038)	0.147	19	509	16	394	1.088 (0.553–2.143)	0.808
		STEMI	266	5128	284	4637	1.756 (1.272–2.424)	0.001	73	2393	102	2616	1.278 (0.942–1.735)	0.115
All bleeding	Age (years)	≥75	75	1202	69	599	2.727 (1.552–4.71)	0.001	19	328	37	303	2.108 (1.186–3.746)	0.011
		< 75	233	5969	262	4782	1.431 (1.089–1.881)	0.010	78	2752	116	2721	1.504 (1.123–2.014)	0.006
	Sex	Male	226	5556	258	4454	1.521 (1.152–2.009)	0.003	77	2479	111	2414	1.480 (1.101–1.990)	0.009
		Female	84	1656	73	957	2.107 (1.236–3.590)	0.006	20	601	42	610	2.069 (1.201–3.566)	0.009
	Renal insufficiency	Yes	123	953	114	655	1.482 (0.941–2.336)	0.090	29	311	54	345	1.679 (1.042–2.703)	0.033
		No	141	3795	159	3315	1.605 (1.150–2.241)	0.005	54	1848	80	1928	1.420 (1.000–2.017)	0.050
	Diabetes	Yes	84	1517	66	1110	1.149 (0.688–1.919)	0.595	28	652	32	630	1.183 (0.704–1.987)	0.526
		No	226	5569	285	4295	1.795 (1.353–2.381)	0.001	69	2428	121	2394	1.779 (1.316–2.403)	<0.001
	Weight (kg)	≥ 60	141	3657	160	2901	1.691 (1.178–2.427)	0.004	42	1666	75	1614	1.843 (1.256–2.706)	0.002
		< 60	73	1253	58	742	1.807 (1.044–3.128)	0.035	27	487	37	436	1.531 (0.917–2.556)	0.104
	Disease status	NSTEMI/UAP	71	1963	32	741	2.449 (1.182–5.075)	0.016	15	696	19	440	2.004 (1.008–3.984)	0.048
		STEMI	228	5166	297	4624	1.557 (1.198–2.024)	0.001	82	2384	134	2584	1.508 (1.139–1.996)	0.004
Major bleeding	Age (years)	≥75	60	1217	46	622	1.620 (0.833–3.152)	0.155	15	332	21	319	1.457 (0.738–2.877)	0.278
		< 75	172	6030	188	4856	1.525 (1.097–2.119)	0.012	50	2780	78	2759	1.572 (1.098–2.251)	0.014
	Sex	Male	168	5614	180	4532	1.451 (1.039–2.028)	0.029	50	2506	70	2455	1.429 (0.990–2.063)	0.057
		Female	66	1074	54	976	2.039 (1.077–3.861)	0.029	15	606	29	623	1.881 (0.998–3.543)	0.051
	Renal insufficiency	Yes	107	969	89	680	1.518 (0.923–2.495)	0.100	21	319	44	355	1.833 (1.096–3.235)	0.022
		No	96	3840	103	3371	1.434 (0.947–2.172)	0.089	35	1867	44	1964	1.195 (0.763–1.871)	0.436
	Diabetes	Yes	64	1537	52	1130	1.222 (0.669–2.231)	0.515	20	660	23	639	1.188 (0.646–2.184)	0.580
		No	170	5751	182	4378	1.699 (1.208–2.391)	0.002	45	2452	76	2439	1.698 (1.169–2.465)	0.005
	Weight (kg)	≥ 60	109	3689	114	2947	1.447 (0.945–8.215)	0.089	30	1678	49	1640	1.671 (1.056–2.646)	0.028
		< 60	45	1281	35	765	1.975 (0.958–4.072)	0.065	16	498	22	451	1.518 (0.788–2.927)	0.212
	Disease status	NSTEMI/UAP	54	1980	18	755	2.344 (0.838–6.560)	0.105	10	701	10	449	1.561 (0.645–3.781)	0.324
		STEMI	171	5223	214	4707	1.518 (1.113–2.009)	0.008	55	2411	89	2629	1.484 (1.056–2.086)	0.023

## Discussion

Our current study was based on the CCC-ACS project and compared outcomes in patients with ACS who received GPI plus ticagrelor or ticagrelor. This large, quantitative study included 13,264 patients across a wide range of characteristics. The findings of this study indicated that the risk of MACE was significantly increased in patients who received GPI plus ticagrelor therapies in the unmatched and PSM analyses. Moreover, the risk of any bleeding and major bleeding were significantly increased in patients receiving combination therapy based on unmatched or matched analyses. For specific adverse events, we noted that combination therapy could affect the risk of all-cause mortality, stent thrombosis, cardiogenic shock, ischemic stroke, and intracranial bleeding. The results of stratified analyses indicated that the effectiveness of combination therapy could be affected by age, sex, renal insufficiency, diabetes, weight, and disease status.

Previous findings demonstrated additional inhibitory effects on platelet aggregation and platelet activation responses by P2Y_12_ inhibitors in the context of GPIs [17]. Numerous observational studies indicated that high platelet reactivity to adenosine diphosphate is an independent risk factor for ischemic events in patients after PCI [18–20]. Moreover, high platelet reactivity is significantly correlated with the extent of atherosclerosis, culprit lesion atherosclerotic disease and adverse plaque morphology in patients undergoing PCI [21, 22]. However, this study found that GPI plus ticagrelor produced excess MACE risk compared to ticagrelor therapy. A possible reason for this result could be that the percentage of STEMI in the GPI plus ticagrelor group was higher than that in the ticagrelor group in unmatched and PSM analyses, which was associated with poor prognosis. Moreover, the use of GPI in patients should be employed for high-risk patients with ACS. The results of stratified analyses suggested that the GPI plus ticagrelor treatment might produce a harmful effect on MACE when patients are less than 75.0 years of age, are male, have renal insufficiency, are without DM, weigh more than 60.0 kg, and have STEMI. These results are based on unmatched analyses, which could bias the conclusions.

Furthermore, we noted that the incidence of stent thrombosis, any bleeding, major bleeding, and intracranial bleeding were significantly higher in the GPI plus ticagrelor group by PSM analysis. A potential reason for the increased risk of stent thrombosis might be that the mean age of patients in the GPI plus ticagrelor group was significantly older than ticagrelor group, which can be associated with a variety of physiological changes and comorbidities, each of which could cause additional risk of stent thrombosis [23]. Moreover, the event of stent thrombosis occurred in GPI plus ticagrelor group and ticagrelor group were smaller than expected, and the results might occasionally. Furthermore, the risk of bleeding events was increased due to inclusion of elderly patients who are more vulnerable to the adverse effects of GPI plus ticagrelor therapies [24]. Finally, although there were no significant differences between the therapies in the incidence of all-cause mortality, MI, cardiogenic shock, ischemic stroke, gastrointestinal bleeding, bleeding requiring surgical intervention, or transfusion with overt bleeding, these results might be impacted by the mean age of patients, DM, type of ACS, cause of hospitalization or other cardiovascular risk factors that were not adjusted for, thus requiring further large-scale studies to verify the above results.

Several strengths of this study should be mentioned. The current study recruited large numbers of patients with ACS across a broad range of characteristics, and this large number of patients provides robust results regarding the efficacy and safety of GPI plus ticagrelor versus ticagrelor therapies. Thus, the results of this study should be recommended for Chinese patients with ACS. Moreover, unmatched and PSM analyses found consistent results for MACE, any bleeding, and major bleeding, demonstrating the consistency of the study’s findings. The limitations of this are as follows: (1) the age and type of ACS remained unbalanced after PSM, which might affect the progression of MACE; (2) stratified analyses based on several important factors were not conducted and the results for secondary outcomes were not evaluated due to lower incidence of interesting outcomes.

## Conclusions

The results of this study suggest that patients with ACS who received GPI plus ticagrelor therapy had a greater risk of in-hospital MACE, any bleeding, and major bleeding, especially for low-risk patients. Moreover, the risk of stent thrombosis, cardiogenic shock, and intracranial bleeding might be increased in patients receiving GPI plus ticagrelor therapy compared to those receiving ticagrelor. Furthermore, combination therapy could improve all-cause mortality and reduce the risk of ischemic stroke. Further large-scale, randomized controlled trials should be conducted to verify the results of this study.

## Supporting information

S1 File Independent predictors of MACE in unmatched cohort and PSM cohort, all bleeding in unmatched cohort and matched cohort and major bleeding in unmatched cohort and matched cohort. (DOCX)
