# Supplementary material for: Combined glycoprotein IIb/IIIa inhibitor therapy with ticagrelor for patients with acute coronary syndrome
Source: PLoS One. 2021 Feb 2;16(2):e0246166. doi: 10.1371/journal.pone.0246166 (PMC7853481; doi:10.1371/journal.pone.0246166)
Supplement: S1 File — (DOCX) [file pone.0246166.s001.docx]

Table S1. Independent Predictors of MACE in unmatched cohort

| Outcomes | OR | CIL | CIU | P value |
| --- | --- | --- | --- | --- |
| Group | 1.669 | 1.249 | 2.231 | 0.001 |
| PCI | 0.762 | 0.335 | 1.736 | 0.518 |
| Culprit vessel |  |  |  |  |
| LM | 0.958 | 0.616 | 1.489 | 0.848 |
| LAD | 2.034 | 0.820 | 5.045 | 0.125 |
| LCX | 0.857 | 0.620 | 1.186 | 0.353 |
| RCA | 0.870 | 0.072 | 10.536 | 0.913 |
| SVG | 0.254 | 0.063 | 1.027 | 0.054 |
| Stent implantation | 0.689 | 0.478 | 0.992 | 0.045 |
| Sex | 0.870 | 0.595 | 1.274 | 0.475 |
| Killip class | | | | |
| I | 1.526 | 1.071 | 2.174 | 0.019 |
| II | 1.279 | 0.641 | 2.554 | 0.485 |
| III-IV | 1.290 | 0.776 | 2.144 | 0.327 |
| Cardiogenic shock | 23.709 | 14.905 | 37.714 | <0.001 |
| Heart failure | 2.091 | 1.426 | 3.066 | <0.001 |
| Sudden cardiac arrest | 1.073 | 0.630 | 1.826 | 0.796 |
| Smoking | 0.820 | 0.593 | 1.133 | 0.229 |
| Previous MI | 0.918 | 0.448 | 1.877 | 0.814 |
| Previous PCI | 1.188 | 0.614 | 2.301 | 0.609 |
| Previous CABG | 4.091 | 0.665 | 25.180 | 0.129 |
| AF history | 1.818 | 0.846 | 3.907 | 0.126 |
| HF history | 0.782 | 0.209 | 2.932 | 0.716 |
| COPD | 1.089 | 0.317 | 3.738 | 0.892 |
| Heart valve surgery history | 0.000 | 0.000 | - | 0.999 |
| PVD history | 1.215 | 0.323 | 4.568 | 0.773 |
| Hypertension | 1.081 | 0.805 | 1.451 | 0.605 |
| Dyslipidemia | 1.144 | 0.658 | 1.991 | 0.633 |
| DM | 0.552 | 0.370 | 0.823 | 0.004 |
| Renal failure history | 1.899 | 0.564 | 6.400 | 0.301 |
| Stroke history | 0.828 | 0.518 | 1.325 | 0.432 |
| STEMI | 0.695 | 0.462 | 1.046 | 0.081 |
| NSTEMI | 0.542 | 0.199 | 1.473 | 0.230 |
| Aspirin | 1.080 | 0.437 | 2.668 | 0.867 |
| β-blocker | 0.852 | 0.618 | 1.173 | 0.325 |
| Warfarin | 0.000 | 0.000 | - | 0.998 |
| ACEI | 1.167 | 0.693 | 1.964 | 0.562 |
| ARB | 1.100 | 0.780 | 1.551 | 0.587 |
| Adosterone receptor antagonist | 1.076 | 0.753 | 1.538 | 0.686 |
| Statins | 1.170 | 0.500 | 2.739 | 0.717 |
| Anticoagulant therapy | 1.043 | 0.723 | 1.505 | 0.821 |
| Serum creatinine (umol/L) | 1.002 | 1.000 | 1.004 | 0.055 |
| Hemoglobin (g/L) | 0.997 | 0.989 | 1.005 | 0.455 |
| FBG (mmol/L) | 1.075 | 1.033 | 1.118 | <0.001 |
| INR | 1.196 | 1.045 | 1.370 | 0.009 |
| TC (mmol/L) | 0.989 | 0.813 | 1.204 | 0.916 |
| HDL (mmol/L) | 0.942 | 0.654 | 1.357 | 0.748 |
| LDL (mmol/L) | 1.075 | 0.855 | 1.353 | 0.535 |
| TG (mmol/L) | 0.973 | 0.852 | 1.111 | 0.684 |
| Age (years) | 1.025 | 1.011 | 1.039 | 0.000 |
| SBP (mmHg) | 0.993 | 0.983 | 1.003 | 0.148 |
| DBP (mmHg) | 0.990 | 0.975 | 1.006 | 0.210 |
| Heart rate | 1.015 | 1.008 | 1.023 | <0.001 |

Table S2. Independent Predictors of MACE in PSM cohort

| Outcomes | OR | CIL | CIU | P value |
| --- | --- | --- | --- | --- |
| Group | 1.505 | 1.086 | 2.084 | 0.014 |
| Sex | 0.930 | 0.606 | 1.428 | 0.740 |
| PCI | 1.374 | 0.368 | 5.122 | 0.636 |
| Culprit vessel | | | | |
| LM | 0.842 | 0.504 | 1.406 | 0.511 |
| LAD | 2.158 | 0.802 | 5.805 | 0.128 |
| LCX | 0.833 | 0.576 | 1.204 | 0.331 |
| RCA | 1.239 | 0.104 | 14.732 | 0.866 |
| SVG | 0.189 | 0.033 | 1.093 | 0.063 |
| Stent implantation | 0.703 | 0.462 | 1.068 | 0.099 |
| Killip class |  |  |  |  |
| I | 1.836 | 1.244 | 2.710 | 0.002 |
| II | 1.241 | 0.555 | 2.776 | 0.599 |
| III-IV | 1.249 | 0.677 | 2.302 | 0.477 |
| Cardiogenic shock | 22.669 | 13.095 | 39.245 | <0.001 |
| Heart failure | 1.723 | 1.097 | 2.706 | 0.018 |
| Sudden cardiac arrest | 0.885 | 0.428 | 1.828 | 0.741 |
| Smoking | 0.831 | 0.577 | 1.196 | 0.318 |
| Previous MI | 0.820 | 0.346 | 1.939 | 0.651 |
| Previous PCI | 1.263 | 0.585 | 2.725 | 0.552 |
| Previous CABG | 5.740 | 0.849 | 38.793 | 0.073 |
| AF history | 1.668 | 0.699 | 3.981 | 0.249 |
| HF history | 1.010 | 0.239 | 4.256 | 0.990 |
| COPD | 0.966 | 0.215 | 4.353 | 0.964 |
| Heart valve surgery history | 0.000 | 0.000 | - | 1.000 |
| PVD history | 0.960 | 0.187 | 4.939 | 0.961 |
| Hypertension | 1.079 | 0.770 | 1.510 | 0.660 |
| Dyslipidemia | 1.106 | 0.560 | 2.183 | 0.772 |
| DM | 0.579 | 0.370 | 0.905 | 0.017 |
| Renal failure history | 1.674 | 0.364 | 7.687 | 0.508 |
| Stroke history | 0.699 | 0.395 | 1.238 | 0.220 |
| STEMI | 0.621 | 0.397 | 0.972 | 0.037 |
| NSTEMI | 0.415 | 0.118 | 1.464 | 0.171 |
| Aspirin | 0.697 | 0.281 | 1.729 | 0.436 |
| β-blocker | 0.772 | 0.537 | 1.111 | 0.164 |
| Warfarin | 0.000 | 0.000 | - | 0.998 |
| ACEI | 1.169 | 0.650 | 2.102 | 0.602 |
| ARB | 1.097 | 0.742 | 1.623 | 0.643 |
| Adosterone receptor antagonist | 1.107 | 0.742 | 1.653 | 0.618 |
| Statins | 1.082 | 0.433 | 2.702 | 0.866 |
| Anticoagulant therapy | 1.120 | 0.738 | 1.700 | 0.595 |
| Serum creatinine (umol/L) | 1.002 | 1.000 | 1.004 | 0.080 |
| Hemoglobin (g/L) | 0.994 | 0.985 | 1.003 | 0.175 |
| FBG (mmol/L) | 1.074 | 1.026 | 1.124 | 0.002 |
| INR | 1.242 | 1.072 | 1.439 | 0.004 |
| Age (years) | 1.027 | 1.010 | 1.044 | 0.001 |
| SBP (mmHg) | 0.995 | 0.984 | 1.006 | 0.346 |
| DBP (mmHg) | 0.989 | 0.971 | 1.006 | 0.200 |
| Heart rate | 1.019 | 1.010 | 1.027 | <0.001 |
| TC (mmol/L) | 0.947 | 0.757 | 1.184 | 0.633 |
| HDL (mmol/L) | 0.719 | 0.425 | 1.216 | 0.218 |
| LDL (mmol/L) | 1.134 | 0.875 | 1.470 | 0.343 |
| TG (mmol/L) | 0.990 | 0.853 | 1.149 | 0.895 |

Table S3. Independent Predictors of all bleeding in unmatched cohort

| Outcomes |  | OR | CIL | CIU | P value |
| --- | --- | --- | --- | --- | --- |
| Group |  | 1.605 | 1.259 | 2.046 | 0.000 |
| Sex |  | 1.089 | 0.785 | 1.510 | 0.610 |
| Age |  | 1.014 | 1.003 | 1.026 | 0.014 |
| Smoking |  | 0.912 | 0.705 | 1.180 | 0.484 |
| previous MI |  | 0.709 | 0.367 | 1.369 | 0.305 |
| previous PCI |  | 1.384 | 0.797 | 2.405 | 0.248 |
| PreviousCABG |  | 3.039 | 0.638 | 14.469 | 0.163 |
| MHAF history |  | 1.519 | 0.759 | 3.040 | 0.237 |
| HF history |  | 0.687 | 0.187 | 2.522 | 0.572 |
| Hypertension |  | 1.182 | 0.925 | 1.510 | 0.182 |
| DM |  | 0.914 | 0.666 | 1.252 | 0.575 |
| Dyslipidemia |  | 1.428 | 0.940 | 2.169 | 0.095 |
| PVD history |  | 3.852 | 1.696 | 8.750 | 0.001 |
| Stroke history |  | 1.073 | 0.733 | 1.571 | 0.715 |
| COPD |  | 1.088 | 0.367 | 3.222 | 0.880 |
| Renal failure history |  | 0.883 | 0.261 | 2.992 | 0.842 |
| Heart valve surgery history |  | — | — |  | 0.999 |
| Type of ACS |  |  |  |  | 0.018 |
|  | STEM | 1.582 | 1.070 | 2.340 | 0.022 |
|  | NSTEMI/UA | 0.617 | 0.233 | 1.634 | 0.332 |
| Heart Rate |  | 1.009 | 1.002 | 1.016 | 0.010 |
| SBP |  | 1.005 | 0.998 | 1.013 | 0.163 |
| DBP |  | 0.995 | 0.983 | 1.007 | 0.436 |
| KILLIP CLASS |  |  |  |  | 0.003 |
|  | KILLIP Ⅰ | 0.736 | 0.527 | 1.029 | 0.073 |
|  | KILLIP Ⅱ | 1.850 | 1.080 | 3.170 | 0.025 |
|  | KILLIP Ⅲ -Ⅳ | 1.794 | 1.018 | 3.162 | 0.043 |
| Cardiogenic shock |  | 1.903 | 1.077 | 3.363 | 0.027 |
| Heart failure |  | 1.658 | 1.120 | 2.453 | 0.011 |
| Sudden cardiac arrest |  | 1.393 | 0.796 | 2.438 | 0.245 |
| PCI |  | 0.693 | 0.329 | 1.458 | 0.333 |
| Culprit vessel |  |  |  |  | 0.140 |
|  | LM | 0.838 | 0.571 | 1.231 | 0.369 |
|  | LAD | 2.289 | 1.033 | 5.071 | 0.041 |
|  | LCX | 0.889 | 0.681 | 1.160 | 0.386 |
|  | RCA | 2.586 | 0.578 | 11.574 | 0.214 |
|  | SVG | 0.759 | 0.340 | 1.690 | 0.499 |
| Stent implantation |  | 0.735 | 0.547 | 0.989 | 0.042 |
| Aspirin |  | 0.388 | 0.215 | 0.698 | 0.002 |
| Statins |  | 1.765 | 0.788 | 3.950 | 0.167 |
| β-blocker |  | 1.050 | 0.820 | 1.344 | 0.701 |
| ARB |  | 0.762 | 0.376 | 2.887 | 0.601 |
| ACEI |  | 0.772 | 0.502 | 1.188 | 0.239 |
| Adosterone receptor antagonist |  | 0.721 | 0.517 | 1.006 | 0.054 |
| Warfarin |  | 3.126 | 1.135 | 8.613 | 0.027 |
| Anticoagulant therapy |  | 1.269 | 0.912 | 1.766 | 0.157 |
| Hemoglobin (g/L) |  | 1.000 | 0.993 | 1.007 | 0.995 |
| Serum creatinine (umol/L) |  | 1.002 | 1.001 | 1.004 | 0.003 |
| FBG (mmol/L) |  | 1.022 | 0.985 | 1.060 | 0.253 |
| INR |  | 1.110 | 0.945 | 1.302 | 0.204 |
| TC |  | 1.362 | 1.118 | 1.659 | 0.002 |
| HDL |  | 0.928 | 0.675 | 1.276 | 0.648 |
| LDL |  | 0.720 | 0.576 | 0.901 | 0.004 |
| TG |  | 0.847 | 0.749 | 0.957 | 0.008 |

Table S4. Independent Predictors of all bleeding in matched cohort

| Outcomes |  | OR | CIL | CIU | P value |
| --- | --- | --- | --- | --- | --- |
| Group |  | 1.631 | 1.244 | 2.139 | 0.000 |
| sex1 |  | 0.917 | 0.636 | 1.322 | 0.643 |
| Age |  | 1.020 | 1.006 | 1.034 | 0.004 |
| Smoking |  | 1.006 | 0.750 | 1.351 | 0.967 |
| previous MI |  | 1.193 | 0.560 | 2.542 | 0.647 |
| previous PCI |  | 0.825 | 0.427 | 1.593 | 0.567 |
| Previous CABG(1) |  | — | — | — | 0.999 |
| AF history |  | 0.457 | 0.222 | 0.940 | 0.033 |
| HF history |  | 1.799 | 0.364 | 8.898 | 0.471 |
| Hypertension |  | 0.949 | 0.715 | 1.260 | 0.719 |
| DM |  | 1.169 | 0.811 | 1.683 | 0.403 |
| Dyslipidemia |  | 0.662 | 0.404 | 1.083 | 0.100 |
| Stroke history |  | 1.020 | 0.652 | 1.598 | 0.930 |
| PVD history |  | 0.396 | 0.135 | 1.160 | 0.091 |
| COPD |  | 0.680 | 0.220 | 2.102 | 0.502 |
| Renal failure history |  | 2.281 | 0.376 | 13.852 | 0.370 |
| Heart valve surgery history |  | — | — | — | 1.000 |
| Type of ACS |  |  |  |  | 0.156 |
|  | STEM | 1.492 | 0.501 | 4.443 | 0.472 |
|  | NSTEMI/UA | 2.081 | 0.723 | 5.989 | 0.174 |
| Heart rate |  | 1.002 | 0.994 | 1.011 | 0.601 |
| SBP |  | 1.001 | 0.988 | 1.016 | 0.836 |
| DBP |  | 1.007 | 0.999 | 1.015 | 0.104 |
| KILLIP CLASS |  |  |  |  | 0.063 |
|  | KILLIP Ⅰ | 0.699 | 0.476 | 1.027 | 0.068 |
|  | KILLIP Ⅱ | 1.487 | 0.762 | 2.899 | 0.245 |
|  | KILLIP Ⅲ -Ⅳ | 1.564 | 0.776 | 3.151 | 0.211 |
| Cardiogenic shock |  | 2.304 | 1.166 | 4.555 | 0.016 |
| Heart failure |  | 1.407 | 0.872 | 2.271 | 0.162 |
| Sudden cardiac arrest |  | 1.993 | 0.985 | 4.033 | 0.055 |
| PCI |  | 0.718 | 0.267 | 1.935 | 0.513 |
| Culprit vessel |  |  |  |  | 0.526 |
|  | LM | 0.917 | 0.599 | 1.404 | 0.690 |
|  | LAD | 2.189 | 0.815 | 5.880 | 0.120 |
|  | LCX | 0.905 | 0.668 | 1.228 | 0.522 |
|  | RCA | 2.205 | 0.272 | 17.872 | 0.459 |
|  | SVG | 0.743 | 0.290 | 1.906 | 0.537 |
| Stent implantation |  | 0.789 | 0.556 | 1.118 | 0.183 |
| Aspirin |  | 0.339 | 0.175 | 0.657 | 0.001 |
| β-blocker |  | 1.143 | 0.862 | 1.516 | 0.354 |
| Warfarin |  | 4.628 | 1.240 | 17.275 | 0.023 |
| ACEI |  | 0.741 | 0.450 | 1.222 | 0.240 |
| ARB |  | 0.502 | 0.274 | 0.918 | 0.025 |
| Adosterone receptor antagonist |  | 0.665 | 0.451 | 0.982 | 0.040 |
| Statins |  | 3.055 | 0.946 | 9.870 | 0.062 |
| Anticoagulant therapy |  | 1.512 | 1.025 | 2.233 | 0.037 |
| Hemoglobin (g/L) |  | 0.993 | 0.985 | 1.000 | 0.057 |
| Serum creatinine (umol/L) |  | 1.003 | 1.001 | 1.005 | 0.003 |
| FBG (mmol/L) |  | 1.048 | 1.004 | 1.093 | 0.031 |
| INR |  | 1.043 | 0.826 | 1.316 | 0.726 |
| TC |  | 1.478 | 1.165 | 1.877 | 0.001 |
| HDL |  | 0.847 | 0.544 | 1.321 | 0.465 |
| LDL |  | 0.678 | 0.520 | 0.884 | 0.004 |
| TG |  | 0.818 | 0.704 | 0.950 | 0.009 |

Table S5. Independent Predictors of major bleeding in unmatched cohort

| Outcomes |  | OR | CIL | CIU | P value |
| --- | --- | --- | --- | --- | --- |
| Group |  | 1.518 | 1.137 | 2.027 | 0.005 |
| sex |  | 0.903 | 0.612 | 1.333 | 0.609 |
| Age |  | 1.014 | 1.001 | 1.028 | 0.041 |
| Smoking |  | 1.040 | 0.762 | 1.418 | 0.806 |
| previous MI |  | 0.843 | 0.395 | 1.801 | 0.660 |
| previous PCI |  | 1.261 | 0.652 | 2.436 | 0.491 |
| PreviousCABG(1) |  | 1.892 | 0.268 | 13.361 | 0.523 |
| AF history |  | 1.221 | 0.519 | 2.872 | 0.648 |
| HF history |  | 0.924 | 0.242 | 3.536 | 0.909 |
| Hypertension |  | 1.267 | 0.944 | 1.700 | 0.114 |
| DM |  | 0.930 | 0.642 | 1.348 | 0.703 |
| Dyslipidemia |  | 1.554 | 0.960 | 2.516 | 0.073 |
| PVD history |  | 3.404 | 1.295 | 8.949 | 0.013 |
| Stroke history |  | 1.036 | 0.660 | 1.626 | 0.879 |
| COPD |  | 0.690 | 0.152 | 3.131 | 0.631 |
| Renal failure history |  | 1.046 | 0.270 | 4.051 | 0.948 |
| Heart valve surgery history |  | — | — | — | 0.999 |
| Type of ACS |  |  |  |  | 0.020 |
|  | STEM | 1.789 | 1.101 | 2.907 | 0.019 |
|  | NSTEMI/UA | 0.589 | 0.169 | 2.055 | 0.407 |
| Heart rate |  | 1.014 | 1.006 | 1.021 | 0.001 |
| SBP |  | 1.011 | 1.002 | 1.020 | 0.017 |
| DBP |  | 0.989 | 0.975 | 1.003 | 0.133 |
| Killip Class |  |  |  |  | 0.025 |
|  | KILLIP Ⅰ | 0.864 | 0.590 | 1.265 | 0.452 |
|  | KILLIP Ⅱ | 2.255 | 1.253 | 4.058 | 0.007 |
|  | KILLIP Ⅲ -Ⅳ | 1.206 | 0.616 | 2.360 | 0.585 |
| Cardiogenic shock |  | 2.604 | 1.377 | 4.925 | 0.003 |
| Heart failure |  | 1.804 | 1.154 | 2.819 | 0.010 |
| Sudden cardiac arrest |  | 1.489 | 0.795 | 2.789 | 0.214 |
| PCI |  | 0.724 | 0.282 | 1.857 | 0.501 |
|  |  |  |  |  | 0.015 |
| Culprit vessel | LM | 0.942 | 0.598 | 1.484 | 0.798 |
|  | LAD | 3.431 | 1.514 | 7.776 | 0.003 |
|  | LCX | 1.013 | 0.736 | 1.392 | 0.939 |
|  | RCA | 4.761 | 1.030 | 22.005 | 0.046 |
|  | SVG | 0.618 | 0.214 | 1.787 | 0.375 |
| Stent implantation |  | 0.692 | 0.490 | 0.977 | 0.036 |
| Aspirin |  | 0.485 | 0.234 | 1.006 | 0.052 |
| Statins |  | 1.519 | 0.627 | 3.680 | 0.354 |
| β-blocker |  | 0.941 | 0.701 | 1.263 | 0.685 |
| ARB |  | 0.577 | 0.354 | 3.076 | 0.614 |
| ACEI |  | 0.796 | 0.477 | 1.330 | 0.384 |
| Adosterone receptor antagonist |  | 0.728 | 0.491 | 1.079 | 0.114 |
| Warfarin |  | 3.828 | 1.244 | 11.783 | 0.019 |
| Anticoagulant therapy |  | 1.184 | 0.802 | 1.747 | 0.395 |
| Hemoglobin (g/L) |  | 1.009 | 1.001 | 1.018 | 0.034 |
| Serum creatinine (umol/L) |  | 1.003 | 1.001 | 1.004 | 0.004 |
| FBG (mmol/L) |  | 1.038 | 0.996 | 1.081 | 0.079 |
| INR |  | 1.153 | 0.979 | 1.357 | 0.088 |
| TC |  | 1.441 | 1.135 | 1.829 | 0.003 |
| HDL |  | 0.946 | 0.654 | 1.369 | 0.769 |
| LDL |  | 0.659 | 0.503 | 0.862 | 0.002 |
| TG |  | 0.777 | 0.664 | 0.910 | 0.002 |

Table S6. Independent Predictors of major bleeding in matched cohort

| Outcomes |  | OR | CIL | CIU | P value |
| --- | --- | --- | --- | --- | --- |
| Group |  | 1.616 | 1.158 | 2.257 | 0.005 |
| Sex |  | 1.192 | 0.759 | 1.873 | 0.445 |
| Age |  | 1.020 | 1.003 | 1.037 | 0.024 |
| Smoking |  | 0.756 | 0.523 | 1.092 | 0.136 |
| previous MI |  | 0.872 | 0.357 | 2.128 | 0.763 |
| previous PCI |  | 1.018 | 0.443 | 2.341 | 0.966 |
| PreviousCABG(1) |  | — | — | — | 0.999 |
| AF history |  | 0.516 | 0.213 | 1.250 | 0.143 |
| HF history |  | 1.339 | 0.251 | 7.147 | 0.732 |
| Hypertension |  | 0.866 | 0.611 | 1.226 | 0.417 |
| DM |  | 1.102 | 0.708 | 1.716 | 0.666 |
| Dyslipidemia |  | 0.607 | 0.338 | 1.092 | 0.096 |
| Stroke history |  | 1.197 | 0.673 | 2.128 | 0.540 |
| PVD history |  | 0.295 | 0.089 | 0.980 | 0.046 |
| COPD |  | 1.196 | 0.242 | 5.904 | 0.826 |
| Renal failure history |  | 3.044 | 0.321 | 28.825 | 0.332 |
| Heart valve surgery history |  | — | — | — | 1.000 |
| Type of ACS |  |  |  |  | 0.197 |
|  | STEM | 1.039 | 0.282 | 3.824 | 0.954 |
|  | NSTEMI | 1.676 | 0.483 | 5.817 | 0.416 |
| Heart rate |  | 1.008 | 0.998 | 1.018 | 0.135 |
| SBP |  | 0.996 | 0.980 | 1.013 | 0.680 |
| DBP |  | 1.015 | 1.005 | 1.024 | 0.002 |
| KILLIP CLASS |  |  |  |  | 0.269 |
|  | KILLIP Ⅰ | 0.796 | 0.506 | 1.251 | 0.322 |
|  | KILLIP Ⅱ | 1.694 | 0.795 | 3.609 | 0.172 |
|  | KILLIP Ⅲ -Ⅳ | 0.808 | 0.340 | 1.918 | 0.628 |
| Cardiogenic shock |  | 3.847 | 1.798 | 8.231 | 0.001 |
| Heart failure |  | 1.552 | 0.885 | 2.722 | 0.125 |
| Sudden cardiac arrest |  | 2.021 | 0.898 | 4.548 | 0.089 |
| PCI |  | 1.031 | 0.233 | 4.570 | 0.968 |
| Culprit vessel |  |  |  |  | 0.149 |
|  | LM | 1.108 | 0.664 | 1.849 | 0.694 |
|  | LAD | 3.339 | 1.199 | 9.301 | 0.021 |
|  | LCX | 1.036 | 0.712 | 1.507 | 0.854 |
|  | RCA | 3.987 | 0.472 | 33.717 | 0.204 |
|  | SVG | 0.479 | 0.113 | 2.039 | 0.320 |
| Stent implantation |  | 0.721 | 0.473 | 1.097 | 0.127 |
| Aspirin |  | 0.398 | 0.173 | 0.918 | 0.031 |
| β-blocker |  | 0.977 | 0.691 | 1.381 | 0.895 |
| Warfarin |  | 4.650 | 0.967 | 22.372 | 0.055 |
| ACEI |  | 0.815 | 0.440 | 1.511 | 0.517 |
| ARB |  | 0.781 | 0.343 | 1.777 | 0.555 |
| Adosterone receptor antagonist |  | 0.624 | 0.384 | 1.012 | 0.056 |
| Statins |  | 3.221 | 0.761 | 13.632 | 0.112 |
| Anticoagulant therapy |  | 1.573 | 0.969 | 2.555 | 0.067 |
| Hemoglobin (g/L) |  | 1.000 | 0.990 | 1.009 | 0.930 |
| Serum creatinine (umol/L) |  | 1.003 | 1.001 | 1.005 | 0.002 |
| FBG (mmol/L) |  | 1.060 | 1.009 | 1.114 | 0.020 |
| INR |  | 1.082 | 0.851 | 1.376 | 0.521 |
| TC |  | 1.753 | 1.320 | 2.330 | 0.000 |
| HDL |  | 0.752 | 0.437 | 1.292 | 0.302 |
| LDL |  | 0.561 | 0.409 | 0.769 | 0.000 |
| TG |  | 0.744 | 0.614 | 0.901 | 0.002 |

Table S7. Independent Predictors of all-cause mortality in unmatched cohort

| Outcomes | OR | CIL | CIU | p valur |
| --- | --- | --- | --- | --- |
| Group | 1.080 | 0.704 | 1.658 | 0.725 |
| PCI | 1.005 | 2.005 | 3.005 | 0.005 |
| Culprit vessel | — | — | — | 0.011 |
| Stent implantation | 2.141 | 1.247 | 3.676 | 0.006 |
| Sex | 0.648 | 0.405 | 1.038 | 0.071 |
| Killip class |  |  |  |  |
| I | 0.923 | 0.291 | 2.929 | 0.892 |
| II | 0.927 | 0.341 | 2.518 | 0.881 |
| III-IV | 1.447 | 0.503 | 4.160 | 0.493 |
| Cardiogenic shock | 5.812 | 3.254 | 10.380 | 0.000 |
| Heart failure | 1.581 | 0.967 | 2.585 | 0.068 |
| Sudden cardiac arrest | 2.355 | 0.983 | 5.328 | 0.045 |
| Smoking | 1.091 | 0.695 | 1.712 | 0.706 |
| Previous MI | 1.137 | 0.457 | 2.828 | 0.782 |
| Previous PCI | 0.457 | 0.158 | 1.318 | 0.147 |
| Previous CABG | — | — | — | 0.998 |
| AF history | 0.759 | 0.304 | 1.894 | 0.555 |
| HF history | 0.731 | 0.256 | 2.085 | 0.558 |
| COPD | 1.189 | 0.262 | 5.401 | 0.823 |
| Heart valve surgery history | — | — | — | 0.999 |
| PVD history | 1.257 | 0.206 | 7.668 | 0.804 |
| Hypertension | 0.738 | 0.490 | 1.113 | 0.147 |
| Dyslipidemia | 3.801 | 1.023 | 14.120 | 0.046 |
| DM | 1.311 | 0.807 | 2.129 | 0.274 |
| Renal failure history | 1.714 | 0.479 | 6.127 | 0.407 |
| Stroke history | 0.944 | 0.544 | 1.640 | 0.839 |
| STEMI | 50.126 | 1.680 | 1495.503 | 0.024 |
| NSTEMI | 6.601 | 0.473 | 92.184 | 0.161 |
| Aspirin | 0.305 | 0.071 | 1.305 | 0.109 |
| β-blocker | 0.982 | 0.630 | 5.843 | 0.935 |
| Warfarin | — | — | — | 0.998 |
| ACEI | 1.125 | 0.534 | 2.367 | 0.757 |
| ARB | 1.052 | 0.650 | 1.702 | 0.836 |
| Adosterone receptor antagonist | 1.544 | 0.938 | 2.542 | 0.088 |
| Statins | 2.157 | 1.069 | 4.350 | 0.032 |
| Anticoagulant therapy | 1.514 | 0.954 | 2.403 | 0.079 |
| Serum creatinine (umol/L) | 1.004 | 1.003 | 1.006 | 0.000 |
| Hemoglobin (g/L) | 1.009 | 0.999 | 1.019 | 0.089 |
| FBG (mmol/L) | 1.169 | 1.122 | 1.217 | 0.000 |
| INR | 1.173 | 1.004 | 1.370 | 0.044 |
| TC (mmol/L) | 1.182 | 0.873 | 1.600 | 0.278 |
| HDL (mmol/L) | 0.942 | 0.601 | 1.476 | 0.795 |
| LDL (mmol/L) | 0.929 | 0.654 | 1.320 | 0.681 |
| TG (mmol/L) | 0.843 | 0.663 | 1.071 | 0.161 |
| Age (years) | 1.065 | 1.044 | 1.086 | 0.000 |
| SBP (mmHg) | 0.983 | 0.971 | 0.996 | 0.010 |
| DBP (mmHg) | 0.997 | 0.976 | 1.018 | 0.752 |
| Heart rate | 1.015 | 1.006 | 1.025 | 0.002 |

Table S8. Independent Predictors of all-cause mortality in matched cohort

| Outcomes | OR | CIL | CIU | p valur |
| --- | --- | --- | --- | --- |
| Group | 0.376 | 0.049 | 2.903 | 0.348 |
| PCI | 7.024 | 0.002 | 611.488 | 0.651 |
| Culprit vessel | — | — | — | 0.668 |
| Stent implantation | 25.112 | 0.008 | 797.622 | 0.432 |
| Sex | 0.788 | 0.087 | 7.171 | 0.832 |
| Killip class | — | — | — | 0.534 |
| Cardiogenic shock | 0.000 | 0.000 |  | 0.994 |
| Heart failure | 18.735 | 0.950 | 369.298 | 0.054 |
| Sudden cardiac arrest | 3.428 | 0.010 | 1219.866 | 0.681 |
| Smoking | 9.903 | 0.541 | 55.033 | 0.969 |
| Previous MI | 0.941 | 0.002 | 544.018 | 0.985 |
| Previous PCI | 0.365 | 0.001 | 130.080 | 0.737 |
| Previous CABG | — | — | — | 0.085 |
| AF history | 12.928 | 0.049 | 3406.796 | 0.368 |
| HF history | 0.561 | 0.278 | 11.032 | 0.771 |
| COPD | 1.928 | 0.041 | 46.597 | 0.997 |
| Heart valve surgery history | — | — | — | 1.000 |
| PVD history | — | — | — | 0.998 |
| Hypertension | 2.435 | 0.276 | 21.497 | 0.423 |
| Dyslipidemia | 2.809 | 0.594 | 13.680 | 0.632 |
| DM | 4.583 | 0.442 | 47.498 | 0.202 |
| Renal failure history | 7.363 | 0.773 | 27.878 | 0.753 |
| Stroke history | — | — | — | 0.991 |
| STEMI | 1.461 | 0.394 | 6.820 | 0.026 |
| NSTEMI | 0.360 | 0.034 | 3.824 | 0.397 |
| Aspirin | 1.693 | 0.694 | 5.104 | 0.017 |
| β-blocker | 1.422 | 0.181 | 11.152 | 0.737 |
| Warfarin | — | — | — | 0.999 |
| ACEI | 1.843 | 0.013 | 268.264 | 0.810 |
| ARB | 0.458 | 0.243 | 6.810 | 0.131 |
| Adosterone receptor antagonist | — | — | — | 0.974 |
| Statins | 0.873 | 0.073 | 0.940 | 0.026 |
| Anticoagulant therapy | 8.944 | 0.067 | 1193.331 | 0.380 |
| Serum creatinine (umol/L) | 0.988 | 0.952 | 1.027 | 0.546 |
| Hemoglobin (g/L) | 0.976 | 0.935 | 1.019 | 0.267 |
| FBG (mmol/L) | 1.039 | 0.773 | 1.395 | 0.802 |
| INR | 0.132 | 0.000 | 107.425 | 0.553 |
| TC (mmol/L) | 3.363 | 0.381 | 29.718 | 0.275 |
| HDL (mmol/L) | 0.009 | 0.000 | 1.178 | 0.058 |
| LDL (mmol/L) | 0.458 | 0.041 | 5.068 | 0.525 |
| TG (mmol/L) | 0.186 | 0.029 | 1.189 | 0.075 |
| Age (years) | 0.954 | 0.862 | 1.056 | 0.364 |
| SBP (mmHg) | 0.929 | 0.849 | 1.017 | 0.112 |
| DBP (mmHg) | 1.053 | 0.925 | 1.198 | 0.436 |
| Heart rate | 0.983 | 0.918 | 1.052 | 0.612 |

Table S9. Independent Predictors of cardiac death in unmatched cohort

| Outcomes | OR | CIL | CIU | p valur |
| --- | --- | --- | --- | --- |
| Group | 1.039 | 0.672 | 1.606 | 0.865 |
| PCI | 0.253 | 0.097 | 0.658 | 0.005 |
| Culprit vessel | — | — | — | 0.017 |
| Stent implantation | 2.084 | 1.204 | 3.608 | 0.009 |
| Sex | 0.674 | 0.419 | 1.085 | 0.105 |
| Killip class |  |  |  |  |
| I | 0.991 | 0.314 | 3.131 | 0.988 |
| II | 0.924 | 0.340 | 2.508 | 0.877 |
| III-IV | 1.437 | 0.499 | 4.143 | 0.502 |
| Cardiogenic shock | 6.185 | 3.459 | 11.060 | 0.000 |
| Heart failure | 1.289 | 0.778 | 2.137 | 0.324 |
| Sudden cardiac arrest | 3.458 | 0.675 | 7.435 | 0.345 |
| Smoking | 1.011 | 0.639 | 1.600 | 0.963 |
| Previous MI | 1.009 | 0.396 | 2.570 | 0.985 |
| Previous PCI | 0.530 | 0.185 | 1.521 | 0.238 |
| Previous CABG | — | — | — | 0.998 |
| AF history | 0.830 | 0.320 | 2.150 | 0.701 |
| HF history | 0.817 | 0.274 | 2.435 | 0.716 |
| COPD | 2.343 | 0.302 | 18.158 | 0.415 |
| Heart valve surgery history | — | — | — | 0.999 |
| PVD history | 1.175 | 0.194 | 7.133 | 0.861 |
| Hypertension | 0.782 | 0.516 | 1.184 | 0.245 |
| Dyslipidemia | 3.698 | 0.992 | 13.789 | 0.051 |
| DM | 1.268 | 0.778 | 2.067 | 0.341 |
| Renal failure history | 1.702 | 0.474 | 6.106 | 0.415 |
| Stroke history | 0.876 | 0.505 | 1.518 | 0.636 |
| STEMI | 39.856 | 1.444 | 1100.248 | 0.029 |
| NSTEMI | 5.151 | 0.412 | 64.453 | 0.204 |
| Aspirin | 0.329 | 0.079 | 1.378 | 0.128 |
| β-blocker | 1.001 | 0.640 | 1.566 | 0.996 |
| Warfarin | — | — | — | 0.997 |
| ACEI | 1.101 | 0.521 | 2.327 | 0.800 |
| ARB | 1.046 | 0.643 | 1.700 | 0.857 |
| Adosterone receptor antagonist | 0.922 | 2.531 | 2.542 | 1.527 |
| Statins | 1.819 | 0.875 | 3.782 | 0.109 |
| Anticoagulant therapy | 1.545 | 0.971 | 2.459 | 0.067 |
| Serum creatinine (umol/L) | 1.004 | 1.003 | 1.006 | 0.000 |
| Hemoglobin (g/L) | 1.006 | 0.996 | 1.017 | 0.250 |
| FBG (mmol/L) | 1.161 | 1.114 | 1.210 | 0.000 |
| INR | 1.175 | 1.006 | 1.373 | 0.042 |
| TC (mmol/L) | 1.196 | 0.879 | 1.628 | 0.255 |
| HDL (mmol/L) | 0.904 | 0.558 | 1.463 | 0.680 |
| LDL (mmol/L) | 0.938 | 0.657 | 1.338 | 0.724 |
| TG (mmol/L) | 0.838 | 0.657 | 1.070 | 0.157 |
| Age (years) | 1.062 | 1.041 | 1.083 | 0.000 |
| SBP (mmHg) | 0.986 | 0.974 | 0.999 | 0.031 |
| DBP (mmHg) | 0.994 | 0.973 | 1.016 | 0.601 |
| Heart rate | 1.017 | 1.007 | 1.027 | 0.000 |

Table S10. Independent Predictors of cardiac death in matched cohort

| Outcomes | OR | CIL | CIU | p valur |
| --- | --- | --- | --- | --- |
| Group | 0.707 | 0.389 | 1.285 | 0.255 |
| PCI | 0.477 | 0.091 | 2.491 | 0.380 |
| Culprit vessel | — | — | — | 0.083 |
| Stent implantation | 0.526 | 0.263 | 1.055 | 0.070 |
| Sex | 0.734 | 0.342 | 1.577 | 0.428 |
| Killip class | — | — | — | 0.270 |
| Cardiogenic shock | 2.749 | 0.984 | 7.683 | 0.054 |
| Heart failure | 1.045 | 0.448 | 2.436 | 0.919 |
| Sudden cardiac arrest | 3.163 | 1.105 | 9.059 | 0.032 |
| Smoking | 1.244 | 0.627 | 2.468 | 0.533 |
| Previous MI | 1.805 | 0.384 | 8.491 | 0.455 |
| Previous PCI | 0.475 | 0.071 | 3.205 | 0.445 |
| Previous CABG | — | — | — | 0.999 |
| AF history | 2.206 | 0.637 | 7.643 | 0.212 |
| HF history | 0.460 | 0.036 | 5.866 | 0.550 |
| COPD | — | — | — | 0.998 |
| Heart valve surgery history | — | — | — | 1.000 |
| PVD history | — | — | — | 0.997 |
| Hypertension | 0.999 | 0.543 | 1.840 | 0.998 |
| Dyslipidemia | 0.466 | 0.061 | 3.546 | 0.461 |
| DM | 0.741 | 0.336 | 1.634 | 0.458 |
| Renal failure history | 2.796 | 0.367 | 21.322 | 0.321 |
| Stroke history | 0.654 | 0.227 | 1.885 | 0.431 |
| STEMI | 3.746 | 1.921 | 7.104 | 0.049 |
| NSTEMI | 0.666 | 0.324 | 1.371 | 0.270 |
| Aspirin | 1.203 | 0.220 | 6.561 | 0.831 |
| β-blocker | 1.026 | 0.552 | 1.910 | 0.934 |
| Warfarin | — | — | — | 0.999 |
| ACEI | 1.491 | 0.407 | 7.919 | 0.485 |
| ARB | — | — | — | 0.994 |
| Adosterone receptor antagonist | 1.032 | 0.493 | 2.159 | 0.933 |
| Statins | 1.061 | 0.254 | 4.435 | 0.935 |
| Anticoagulant therapy | 0.633 | 0.314 | 1.276 | 0.201 |
| Serum creatinine (umol/L) | 1.003 | 0.999 | 1.006 | 0.116 |
| Hemoglobin (g/L) | 1.003 | 0.987 | 1.019 | 0.727 |
| FBG (mmol/L) | 1.141 | 1.067 | 1.221 | 0.000 |
| INR | 1.352 | 1.143 | 1.598 | 0.000 |
| TC (mmol/L) | 1.497 | 0.866 | 2.587 | 0.148 |
| HDL (mmol/L) | 0.608 | 0.224 | 1.652 | 0.329 |
| LDL (mmol/L) | 0.681 | 0.370 | 1.254 | 0.218 |
| TG (mmol/L) | 0.769 | 0.516 | 1.145 | 0.195 |
| Age (years) | 1.065 | 1.033 | 1.098 | 0.000 |
| SBP (mmHg) | 0.993 | 0.974 | 1.012 | 0.481 |
| DBP (mmHg) | 0.977 | 0.945 | 1.010 | 0.176 |
| Heart rate | 1.019 | 1.004 | 1.034 | 0.014 |

Table S11. Independent Predictors of MI in unmatched cohort

| Outcomes | OR | CIL | CIU | p valur |
| --- | --- | --- | --- | --- |
| Group | 0.632 | 0.273 | 1.465 | 0.285 |
| PCI | 0.591 | 0.063 | 5.568 | 0.646 |
| Culprit vessel | — | — | — | 0.662 |
| Stent implantation | 1.574 | 0.533 | 4.647 | 0.411 |
| Sex | 0.727 | 0.286 | 1.848 | 0.503 |
| Killip class | — | — | — | 0.361 |
| Cardiogenic shock | 3.432 | 0.843 | 13.543 | 0.031 |
| Heart failure | 2.991 | 0.984 | 9.091 | 0.053 |
| Sudden cardiac arrest | 0.807 | 0.091 | 7.128 | 0.847 |
| Smoking | 0.647 | 0.271 | 1.544 | 0.326 |
| Previous MI | 10.929 | 2.900 | 41.188 | 0.000 |
| Previous PCI | 0.497 | 0.112 | 2.211 | 0.358 |
| Previous CABG | — | — | — | 0.998 |
| AF history | — | — | — | 0.995 |
| HF history | 0.456 | 0.077 | 2.704 | 0.387 |
| COPD | 0.430 | 0.050 | 3.703 | 0.443 |
| Heart valve surgery history | — | — | — | 0.999 |
| PVD history | 0.350 | 0.023 | 5.372 | 0.452 |
| Hypertension | 1.626 | 0.717 | 3.684 | 0.244 |
| Dyslipidemia | 0.631 | 0.166 | 2.401 | 0.499 |
| DM | 9.823 | 1.957 | 49.322 | 0.006 |
| Renal failure history | 0.435 | 0.030 | 6.370 | 0.543 |
| Stroke history | 1.277 | 0.341 | 4.787 | 0.717 |
| STEMI | 3.856 | 1.644 | 11.248 | 0.021 |
| NSTEMI | 4.131 | 0.611 | 14.453 | 0.203 |
| Aspirin | 1.075 | 0.576 | 1.290 | 0.111 |
| β-blocker | 0.728 | 0.311 | 1.702 | 0.464 |
| Warfarin | — | — | — | 0.997 |
| ACEI | 0.338 | 0.118 | 0.972 | 0.044 |
| ARB | 0.787 | 0.331 | 1.873 | 0.589 |
| Adosterone receptor antagonist | 0.964 | 0.383 | 2.428 | 0.938 |
| Statins | 0.411 | 0.023 | 7.375 | 0.546 |
| Anticoagulant therapy | 1.457 | 0.591 | 3.588 | 0.414 |
| Serum creatinine (umol/L) | 1.002 | 0.999 | 1.006 | 0.157 |
| Hemoglobin (g/L) | 0.990 | 0.970 | 1.012 | 0.376 |
| FBG (mmol/L) | 1.078 | 0.957 | 1.216 | 0.217 |
| INR | 0.855 | 0.486 | 1.506 | 0.587 |
| TC (mmol/L) | 1.560 | 0.884 | 2.751 | 0.125 |
| HDL (mmol/L) | 0.760 | 0.246 | 2.350 | 0.633 |
| LDL (mmol/L) | 0.485 | 0.247 | 0.954 | 0.036 |
| TG (mmol/L) | 1.137 | 0.854 | 1.516 | 0.379 |
| Age (years) | 1.049 | 1.010 | 1.089 | 0.013 |
| SBP (mmHg) | 1.004 | 0.980 | 1.029 | 0.729 |
| DBP (mmHg) | 0.988 | 0.948 | 1.029 | 0.558 |
| Heart rate | 1.019 | 0.998 | 1.041 | 0.074 |

Table S12. Independent Predictors of MI in matched cohort

| Outcomes | p valur | OR | CIL | CIU |
| --- | --- | --- | --- | --- |
| Group | .696 | .819 | .302 | 2.226 |
| PCI | .692 | .630 | .064 | 6.188 |
| Culprit vessel | .581 |  |  |  |
| Stent implantation | .409 | .596 | .174 | 2.039 |
| Sex | .804 | .859 | .257 | 2.865 |
| Killip class | .972 |  |  |  |
| Cardiogenic shock | .994 | .000 | 0.000 |  |
| Heart failure | .157 | 2.861 | .668 | 12.255 |
| Sudden cardiac arrest | .996 | .000 | 0.000 |  |
| Smoking | .716 | .807 | .255 | 2.556 |
| Previous MI | .008 | 9.512 | 1.787 | 50.635 |
| Previous PCI | .545 | .553 | .081 | 3.763 |
| Previous CABG | .998 | .000 | 0.000 |  |
| AF history | .995 | .000 | 0.000 |  |
| HF history | .419 | 2.982 | .210 | 42.265 |
| COPD | .998 | .000 | 0.000 |  |
| Heart valve surgery history | .999 | 3195395609301530.000 | 0.000 |  |
| PVD history | .998 | .000 | 0.000 |  |
| Hypertension | .797 | .867 | .293 | 2.567 |
| Dyslipidemia | .272 | 2.278 | .524 | 9.906 |
| DM | .034 | .133 | .021 | .858 |
| Renal failure history | .086 | 15.116 | .678 | 336.904 |
| Stroke history | .898 | .897 | .169 | 4.766 |
| STEMI |  |  |  |  |
| NSTEMI | .683 | 1.239 | .443 | 3.462 |
| Aspirin |  |  |  |  |
| β-blocker | .843 | 1.110 | .396 | 3.113 |
| Warfarin | .999 |  |  |  |
| ACEI | .121 | 3.757 | .704 | 20.033 |
| ARB | .994 |  |  |  |
| Adosterone receptor antagonist | .337 | 1.779 | .548 | 5.772 |
| Statins | .995 | 3458540.245 |  |  |
| Anticoagulant therapy | .756 | .815 | .225 | 2.949 |
| Serum creatinine (umol/L) | .282 | 1.003 | .998 | 1.007 |
| Hemoglobin (g/L) | .128 | .980 | .954 | 1.006 |
| FBG (mmol/L) | .677 | 1.037 | .874 | 1.230 |
| INR | .905 | .933 | .299 | 2.908 |
| TC (mmol/L) | .098 | 1.656 | .911 | 3.010 |
| HDL (mmol/L) | .734 | .772 | .173 | 3.438 |
| LDL (mmol/L) | .026 | .427 | .202 | .903 |
| TG (mmol/L) | .279 | 1.176 | .877 | 1.578 |
| Age (years) | .013 | 1.070 | 1.014 | 1.130 |
| SBP (mmHg) | .298 | 1.015 | .987 | 1.044 |
| DBP (mmHg) | .918 | .997 | .949 | 1.048 |
| Heart rate | .144 | 1.024 | .992 | 1.057 |

Table S13. Independent Predictors of stent thrombosis in unmatched cohort

| Outcomes | OR | CIL | CIU | p valur |
| --- | --- | --- | --- | --- |
| Group | 7.469 | 1.985 | 28.108 | 0.003 |
| Culprit vessel | — | — | — | 0.969 |
| Sex | 0.691 | 0.202 | 2.358 | 0.555 |
| Killip class |  |  |  |  |
| I | 0.141 | 0.008 | 2.634 | 0.189 |
| II | 1.053 | 0.063 | 17.685 | 0.971 |
| III-IV | 1.075 | 0.132 | 8.764 | 0.946 |
| Cardiogenic shock | 3.049 | 0.537 | 17.320 | 0.208 |
| Heart failure | 3.992 | 1.138 | 14.006 | 0.031 |
| Sudden cardiac arrest | — | — | — | 0.965 |
| Smoking | 0.301 | 0.110 | 0.820 | 0.019 |
| Previous MI | 0.573 | 0.063 | 5.203 | 0.621 |
| Previous PCI | 1.995 | 0.358 | 11.110 | 0.430 |
| Previous CABG | — | — | — | 0.998 |
| AF history | 0.446 | 0.035 | 5.617 | 0.532 |
| HF history | 0.248 | 0.013 | 4.690 | 0.352 |
| COPD | 4.345 | 0.342 | 8.546 | 0.697 |
| Heart valve surgery history | — | — | — | 0.997 |
| PVD history | — | — | — | 0.998 |
| Hypertension | 1.278 | 0.512 | 3.187 | 0.599 |
| Dyslipidemia | 0.235 | 0.077 | 0.723 | 0.012 |
| DM | 2.073 | 0.591 | 7.267 | 0.255 |
| Renal failure history | 1.354 | 0.224 | 7.143 | 0.697 |
| Stroke history | 3.249 | 0.354 | 29.814 | 0.297 |
| STEMI | 0.106 | 0.010 | 1.135 | 0.064 |
| NSTEMI | 0.499 | 0.029 | 8.689 | 0.633 |
| Aspirin | 1.886 | 0.926 | 7.103 | 0.828 |
| β-blocker | 0.319 | 0.101 | 1.012 | 0.053 |
| Warfarin | — | — | — | 0.998 |
| ACEI | 2.133 | 0.242 | 18.834 | 0.496 |
| ARB | 0.747 | 0.284 | 1.962 | 0.553 |
| Adosterone receptor antagonist | 1.539 | 0.458 | 5.171 | 0.486 |
| Statins | 0.682 | 0.840 | 5.080 | 0.253 |
| Anticoagulant therapy | 0.474 | 0.094 | 2.397 | 0.367 |
| Serum creatinine (umol/L) | 0.991 | 0.970 | 1.012 | 0.383 |
| Hemoglobin (g/L) | 1.013 | 0.984 | 1.043 | 0.392 |
| FBG (mmol/L) | 1.054 | 0.922 | 1.204 | 0.441 |
| INR | 0.810 | 0.230 | 2.851 | 0.743 |
| TC (mmol/L) | 1.678 | 0.882 | 3.192 | 0.115 |
| HDL (mmol/L) | 0.258 | 0.042 | 1.588 | 0.144 |
| LDL (mmol/L) | 0.604 | 0.289 | 1.265 | 0.181 |
| TG (mmol/L) | 0.998 | 0.725 | 1.372 | 0.988 |
| Age (years) | 1.018 | 0.975 | 1.062 | 0.419 |
| SBP (mmHg) | 1.002 | 0.972 | 1.033 | 0.896 |
| DBP (mmHg) | 0.970 | 0.925 | 1.017 | 0.207 |
| Heart rate | 1.005 | 0.978 | 1.032 | 0.730 |

Table S14. Independent Predictors of stent thrombosis in matched cohort

| Outcomes | OR | CIL | CIU | p valur |
| --- | --- | --- | --- | --- |
| Group | 7.111 | 1.614 | 31.340 | 0.010 |
| PCI | 0.726 | 0.575 | 2.111 | 0.606 |
| Culprit vessel | — | — | — | 0.665 |
| Stent implantation | 0.539 | 0.131 | 2.224 | 0.393 |
| Sex | 0.501 | 0.114 | 2.199 | 0.360 |
| Killip class | — | — | — | 0.113 |
| Cardiogenic shock | 15.169 | 1.434 | 160.429 | 0.024 |
| Heart failure | 3.032 | 0.472 | 19.459 | 0.242 |
| Sudden cardiac arrest | — | — | — | 0.997 |
| Smoking | 0.463 | 0.118 | 1.821 | 0.270 |
| Previous MI | 0.204 | 0.011 | 3.718 | 0.283 |
| Previous PCI | 2.194 | 0.297 | 16.211 | 0.441 |
| Previous CABG | — | — | — | 0.999 |
| AF history | 1.114 | 0.047 | 26.368 | 0.947 |
| HF history | 20.335 | 0.629 | 657.635 | 0.089 |
| COPD | 1.397 | 0.610 | 6.544 | 0.760 |
| Heart valve surgery history | — | — | — | 1.000 |
| PVD history | — | — | — | 0.998 |
| Hypertension | 0.617 | 0.189 | 2.006 | 0.422 |
| Dyslipidemia | 8.387 | 2.226 | 31.598 | 0.002 |
| DM | 0.581 | 0.130 | 2.605 | 0.478 |
| Renal failure history | 1.292 | 0.855 | 5.659 | 0.889 |
| Stroke history | — | — | — | 0.991 |
| STEMI | 5.102 | 1.049 | 24.810 | 0.043 |
| NSTEMI | 1.185 | 0.410 | 8.097 | 0.095 |
| Aspirin | — | — | — | 0.996 |
| β-blocker | 4.748 | 1.046 | 21.545 | 0.044 |
| Warfarin | — | — | — | 0.999 |
| ACEI | 1.234 | 0.100 | 15.264 | 0.870 |
| ARB | — | — | — | 0.994 |
| Adosterone receptor antagonist | 0.641 | 0.133 | 3.094 | 0.580 |
| Statins | 0.510 | 0.152 | 9.713 | 0.640 |
| Anticoagulant therapy | 1.931 | 0.352 | 10.609 | 0.449 |
| Serum creatinine (umol/L) | 0.974 | 0.945 | 1.005 | 0.099 |
| Hemoglobin (g/L) | 1.020 | 0.983 | 1.058 | 0.294 |
| FBG (mmol/L) | 1.058 | 0.884 | 1.267 | 0.539 |
| INR | 1.010 | 0.311 | 3.280 | 0.987 |
| TC (mmol/L) | 1.363 | 0.674 | 2.757 | 0.389 |
| HDL (mmol/L) | 0.321 | 0.038 | 2.678 | 0.294 |
| LDL (mmol/L) | 0.501 | 0.208 | 1.203 | 0.122 |
| TG (mmol/L) | 1.046 | 0.750 | 1.459 | 0.791 |
| Age (years) | 1.032 | 0.972 | 1.095 | 0.310 |
| SBP (mmHg) | 1.011 | 0.974 | 1.049 | 0.578 |
| DBP (mmHg) | 0.962 | 0.905 | 1.021 | 0.204 |
| Heart rate | 1.036 | 1.000 | 1.073 | 0.050 |

Table S15. Independent Predictors of cardiogenic shock in unmatched cohort

| Outcomes | OR | CIL | CIU | p valur |
| --- | --- | --- | --- | --- |
| Group | 1.529 | 1.156 | 2.023 | 0.003 |
| PCI | 0.924 | 0.412 | 2.074 | 0.849 |
| Culprit vessel | — | — | — | 0.998 |
| Stent implantation | 1.468 | 1.013 | 2.127 | 0.043 |
| Sex | 0.911 | 0.638 | 1.302 | 0.611 |
| Killip class |  |  |  |  |
| I | 1.507 | 0.641 | 3.545 | 0.347 |
| II | 0.887 | 0.418 | 1.885 | 0.756 |
| III-IV | 5.523 | 2.603 | 11.720 | 0.000 |
| Cardiogenic shock | 2.306 | 1.489 | 3.571 | 0.000 |
| Heart failure | 3.584 | 2.577 | 4.984 | 0.000 |
| Sudden cardiac arrest | — | — | — | 0.992 |
| Smoking | 1.043 | 0.772 | 1.408 | 0.785 |
| Previous MI | 1.125 | 0.591 | 2.140 | 0.720 |
| Previous PCI | 0.965 | 0.510 | 1.824 | 0.912 |
| Previous CABG | 0.639 | 0.061 | 6.728 | 0.709 |
| AF history | 0.762 | 0.335 | 1.735 | 0.518 |
| HF history | 2.073 | 0.803 | 5.356 | 0.132 |
| COPD | 0.487 | 0.197 | 1.200 | 0.118 |
| Heart valve surgery history | — | — | — | 0.999 |
| PVD history | 0.677 | 0.212 | 2.162 | 0.510 |
| Hypertension | 0.997 | 0.755 | 1.317 | 0.984 |
| Dyslipidemia | 0.800 | 0.458 | 1.396 | 0.432 |
| DM | 1.403 | 0.989 | 1.990 | 0.058 |
| Renal failure history | 0.972 | 0.322 | 2.933 | 0.960 |
| Stroke history | 0.939 | 0.624 | 1.414 | 0.765 |
| STEMI | 18.126 | 3.680 | 45.766 | 0.039 |
| NSTEMI | 3.230 | 0.706 | 18.365 | 0.295 |
| Aspirin | 1.167 | 0.528 | 2.577 | 0.703 |
| β-blocker | 1.685 | 1.231 | 2.306 | 0.001 |
| Warfarin | — | — | — | 0.997 |
| ACEI | 1.177 | 0.674 | 2.055 | 0.567 |
| ARB | 1.221 | 0.860 | 1.733 | 0.264 |
| Adosterone receptor antagonist | 1.074 | 0.767 | 1.503 | 0.678 |
| Statins | 0.426 | 0.190 | 0.954 | 0.038 |
| Anticoagulant therapy | 0.971 | 0.679 | 1.388 | 0.871 |
| Serum creatinine (umol/L) | 1.002 | 1.000 | 1.004 | 0.034 |
| Hemoglobin (g/L) | 0.996 | 0.988 | 1.003 | 0.269 |
| FBG (mmol/L) | 1.083 | 1.045 | 1.122 | 0.000 |
| INR | 1.175 | 1.035 | 1.334 | 0.013 |
| TC (mmol/L) | 0.890 | 0.745 | 1.064 | 0.200 |
| HDL (mmol/L) | 0.873 | 0.630 | 1.211 | 0.417 |
| LDL (mmol/L) | 1.125 | 0.911 | 1.390 | 0.274 |
| TG (mmol/L) | 0.960 | 0.841 | 1.096 | 0.547 |
| Age (years) | 1.021 | 1.008 | 1.034 | 0.002 |
| SBP (mmHg) | 0.987 | 0.978 | 0.996 | 0.006 |
| DBP (mmHg) | 0.980 | 0.965 | 0.995 | 0.010 |
| Heart rate | 1.015 | 1.008 | 1.022 | 0.000 |

Table S16. Independent Predictors of cardiogenic shock in matched cohort

| Outcomes | OR | CIL | CIU | p valur |
| --- | --- | --- | --- | --- |
| Group | 1.703 | 1.132 | 2.563 | 0.011 |
| PCI | 2.448 | 0.312 | 19.181 | 0.394 |
| Culprit vessel | — | — | — | 0.014 |
| Stent implantation | 0.596 | 0.355 | 1.000 | 0.050 |
| Sex | 1.150 | 0.665 | 1.989 | 0.616 |
| Killip class | — | — | — | 0.141 |
| Cardiogenic shock | 36.608 | 20.123 | 66.597 | 0.000 |
| Heart failure | 1.852 | 1.094 | 3.133 | 0.022 |
| Sudden cardiac arrest | 0.449 | 0.189 | 1.070 | 0.071 |
| Smoking | 0.855 | 0.543 | 1.347 | 0.499 |
| Previous MI | 0.413 | 0.127 | 1.338 | 0.140 |
| Previous PCI | 1.055 | 0.384 | 2.901 | 0.917 |
| Previous CABG | 8.725 | 0.747 | 101.860 | 0.084 |
| AF history | 0.971 | 0.289 | 3.262 | 0.963 |
| HF history | 1.933 | 0.362 | 10.332 | 0.441 |
| COPD | 1.786 | 0.371 | 8.595 | 0.470 |
| Heart valve surgery history | — | — | — | 1.000 |
| PVD history | 1.082 | 0.185 | 6.335 | 0.930 |
| Hypertension | 1.350 | 0.885 | 2.060 | 0.164 |
| Dyslipidemia | 0.737 | 0.268 | 2.026 | 0.554 |
| DM | 0.603 | 0.347 | 1.048 | 0.073 |
| Renal failure history | 0.858 | 0.135 | 5.441 | 0.871 |
| Stroke history | 0.862 | 0.443 | 1.678 | 0.662 |
| STEMI | 0.482 | 0.293 | 0.795 | 0.004 |
| NSTEMI | 0.474 | 0.925 | 4.549 | 0.208 |
| Aspirin | 0.433 | 0.158 | 1.189 | 0.105 |
| β-blocker | 0.530 | 0.346 | 0.811 | 0.004 |
| Warfarin | — | — | — | 0.998 |
| ACEI | 1.935 | 0.676 | 5.543 | 0.219 |
| ARB | 1.520 | 0.421 | 5.491 | 0.523 |
| Adosterone receptor antagonist | 1.023 | 0.617 | 1.696 | 0.930 |
| Statins | 3.272 | 0.761 | 14.067 | 0.111 |
| Anticoagulant therapy | 1.355 | 0.791 | 2.321 | 0.268 |
| Serum creatinine (umol/L) | 1.003 | 1.000 | 1.005 | 0.037 |
| Hemoglobin (g/L) | 0.989 | 0.979 | 0.999 | 0.035 |
| FBG (mmol/L) | 1.064 | 1.006 | 1.126 | 0.029 |
| INR | 1.335 | 1.137 | 1.568 | 0.000 |
| TC (mmol/L) | 0.815 | 0.630 | 1.055 | 0.121 |
| HDL (mmol/L) | 0.611 | 0.310 | 1.203 | 0.154 |
| LDL (mmol/L) | 1.327 | 0.996 | 1.768 | 0.053 |
| TG (mmol/L) | 0.962 | 0.790 | 1.172 | 0.701 |
| Age (years) | 1.022 | 1.001 | 1.043 | 0.044 |
| SBP (mmHg) | 0.997 | 0.984 | 1.011 | 0.668 |
| DBP (mmHg) | 0.986 | 0.965 | 1.008 | 0.221 |
| Heart rate | 1.019 | 1.009 | 1.029 | 0.000 |

Table S17. Independent Predictors of ischemic stroke in unmatched cohort

| Outcomes | OR | CIL | CIU | p valur |
| --- | --- | --- | --- | --- |
| Group | 0.448 | 0.128 | 1.565 | 0.208 |
| PCI | 0.175 | 0.017 | 1.787 | 0.141 |
| Culprit vessel | — | — | — | 0.999 |
| LM | 0.058 | 0.001 | 4.599 | 0.202 |
| LAD | 0.035 | 0.001 | 2.369 | 0.119 |
| LCX | 0.090 | 0.001 | 6.656 | 0.273 |
| RCA | 0.245 | 0.003 | 22.870 | 0.543 |
| SVG | 0.027 | 0.000 | 1.879 | 0.095 |
| Stent implantation | 0.251 | 0.025 | 2.505 | 0.239 |
| Sex | 0.474 | 0.141 | 1.592 | 0.227 |
| Killip class |  |  |  |  |
| I | 0.619 | 0.064 | 5.964 | 0.678 |
| II | 0.510 | 0.084 | 3.110 | 0.465 |
| III-IV | 0.448 | 0.083 | 2.408 | 0.350 |
| Cardiogenic shock | 4.811 | 0.660 | 35.055 | 0.121 |
| Heart failure | 4.739 | 1.223 | 18.367 | 0.024 |
| Sudden cardiac arrest | — | — | — | 0.998 |
| Smoking | 0.689 | 0.203 | 2.334 | 0.549 |
| Previous MI | 0.095 | 0.002 | 3.813 | 0.211 |
| Previous PCI | 1.333 | 0.170 | 10.469 | 0.784 |
| Previous CABG | 0.105 | 0.003 | 3.915 | 0.223 |
| AF history | 0.213 | 0.042 | 1.070 | 0.060 |
| HF history | 1.547 | 0.984 | 4.694 | 0.137 |
| COPD | 2.417 | 0.882 | 5.307 | 0.359 |
| Heart valve surgery history | — | — | — | 0.257 |
| PVD history | — | — | — | 0.976 |
| Hypertension | 0.218 | 0.071 | 0.670 | 0.008 |
| Dyslipidemia | 0.986 | 0.719 | 6.168 | 0.620 |
| DM | 0.718 | 0.191 | 2.692 | 0.623 |
| Renal failure history | 3.140 | 0.894 | 8.549 | 0.458 |
| Stroke history | 0.675 | 0.169 | 2.702 | 0.579 |
| STEMI | 0.106 | 0.009 | 1.209 | 0.071 |
| NSTEMI | 0.082 | 0.008 | 0.830 | 0.034 |
| Aspirin | 4.500 | 0.478 | 42.323 | 0.188 |
| β-blocker | 0.751 | 0.249 | 2.261 | 0.610 |
| Warfarin | — | — | — | 0.133 |
| ACEI | 0.228 | 0.064 | 0.818 | 0.023 |
| ARB | 1.047 | 0.307 | 3.568 | 0.942 |
| Adosterone receptor antagonist | 2.387 | 0.566 | 10.060 | 0.236 |
| Statins | 0.355 | 0.028 | 4.474 | 0.423 |
| Anticoagulant therapy | 1.458 | 0.471 | 4.509 | 0.513 |
| Serum creatinine (umol/L) | 1.001 | 0.988 | 1.013 | 0.930 |
| Hemoglobin (g/L) | 0.999 | 0.972 | 1.026 | 0.918 |
| FBG (mmol/L) | 0.834 | 0.649 | 1.072 | 0.156 |
| INR | 1.393 | 1.155 | 1.681 | 0.001 |
| TC (mmol/L) | 1.024 | 0.414 | 2.531 | 0.959 |
| HDL (mmol/L) | 0.377 | 0.070 | 2.033 | 0.257 |
| LDL (mmol/L) | 1.130 | 0.419 | 3.046 | 0.810 |
| TG (mmol/L) | 0.218 | 0.071 | 0.670 | 0.008 |
| Age (years) | 1.016 | 0.968 | 1.066 | 0.513 |
| SBP (mmHg) | 0.990 | 0.958 | 1.024 | 0.570 |
| DBP (mmHg) | 1.005 | 0.953 | 1.059 | 0.861 |
| Heart rate | 1.026 | 0.997 | 1.055 | 0.076 |

Table S18. Independent Predictors of ischemic stroke in matched cohort

| Outcomes | OR | CIL | CIU | p valur |
| --- | --- | --- | --- | --- |
| Group | 0.376 | 0.049 | 2.903 | 0.348 |
| PCI | 7.024 | 0.002 | 611.488 | 0.651 |
| Culprit vessel | — | — | — | 0.668 |
| Stent implantation | 25.112 | 0.008 | 797.622 | 0.432 |
| Sex | 0.788 | 0.087 | 7.171 | 0.832 |
| Killip class | — | — | — | 0.534 |
| Cardiogenic shock | 0.000 | 0.000 |  | 0.994 |
| Heart failure | 18.735 | 0.950 | 369.298 | 0.054 |
| Sudden cardiac arrest | 3.428 | 0.010 | 1219.866 | 0.681 |
| Smoking | 9.903 | 0.541 | 55.033 | 0.969 |
| Previous MI | 0.941 | 0.002 | 544.018 | 0.985 |
| Previous PCI | 0.365 | 0.001 | 130.080 | 0.737 |
| Previous CABG | — | — | — | 0.085 |
| AF history | 12.928 | 0.049 | 3406.796 | 0.368 |
| HF history | 0.561 | 0.278 | 11.032 | 0.771 |
| COPD | 1.928 | 0.041 | 46.597 | 0.997 |
| Heart valve surgery history | — | — | — | 1.000 |
| PVD history | — | — | — | 0.998 |
| Hypertension | 2.435 | 0.276 | 21.497 | 0.423 |
| Dyslipidemia | 2.809 | 0.594 | 13.680 | 0.632 |
| DM | 4.583 | 0.442 | 47.498 | 0.202 |
| Renal failure history | 7.363 | 0.773 | 27.878 | 0.753 |
| Stroke history | — | — | — | 0.991 |
| STEMI | 1.461 | 0.394 | 6.820 | 0.026 |
| NSTEMI | 0.360 | 0.034 | 3.824 | 0.397 |
| Aspirin | 1.693 | 0.694 | 5.104 | 0.017 |
| β-blocker | 1.422 | 0.181 | 11.152 | 0.737 |
| Warfarin | — | — | — | 0.999 |
| ACEI | 1.843 | 0.013 | 268.264 | 0.810 |
| ARB | 0.458 | 0.243 | 6.810 | 0.131 |
| Adosterone receptor antagonist | — | — | — | 0.974 |
| Statins | 0.873 | 0.073 | 0.940 | 0.026 |
| Anticoagulant therapy | 8.944 | 0.067 | 1193.331 | 0.380 |
| Serum creatinine (umol/L) | 0.988 | 0.952 | 1.027 | 0.546 |
| Hemoglobin (g/L) | 0.976 | 0.935 | 1.019 | 0.267 |
| FBG (mmol/L) | 1.039 | 0.773 | 1.395 | 0.802 |
| INR | 0.132 | 0.000 | 107.425 | 0.553 |
| TC (mmol/L) | 3.363 | 0.381 | 29.718 | 0.275 |
| HDL (mmol/L) | 0.009 | 0.000 | 1.178 | 0.058 |
| LDL (mmol/L) | 0.458 | 0.041 | 5.068 | 0.525 |
| TG (mmol/L) | 0.186 | 0.029 | 1.189 | 0.075 |
| Age (years) | 0.954 | 0.862 | 1.056 | 0.364 |
| SBP (mmHg) | 0.929 | 0.849 | 1.017 | 0.112 |
| DBP (mmHg) | 1.053 | 0.925 | 1.198 | 0.436 |
| Heart rate | 0.983 | 0.918 | 1.052 | 0.612 |
